# Immunosenescence: Molecular Mechanisms, Diseases, and Therapeutic Innovations

**DOI:** 10.1002/mco2.70515

**Published:** 2025-12-18

**Authors:** Ninghan Gong, Xiting Pan, Yusi Deng, Jiajia Che, Junhao Bao, Mengqi Wang, Chuan Xu, Xiaowei Liu, Ying Shi

**Affiliations:** ^1^ Department of Oncology Sichuan Academy of Medical Sciences Sichuan Provincial People's Hospital School of Medicine University of Electronic Science and Technology of China Chengdu China; ^2^ Institute for Breast Health Medicine, State Key Laboratory of Biotherapy West China Hospital, Sichuan University Chengdu China

**Keywords:** chronic inflammation, hypoxia, immunosenescence, immunotherapy, SASP, tumor‐induced senescence

## Abstract

Immunosenescence denotes progressive deterioration of immune system during physiological aging, initially recognized by the observation of heightened susceptibility to diverse pathologies in elder population. Beyond exhibiting canonical cellular senescence features, senescent immune cells manifest multidimensional dysfunction characterized by impaired secretory capacity and functional disorders. This process further triggers systemic epigenetic dysregulation and failure in damage repair, which collectively remodel metabolic and inflammatory microenvironments to attenuate immune responses and elevate risks of diverse degenerative diseases or multiple types of cancer. Critically, senescence‐associated secretory phenotype (SASP) factors secreted by senescent cells display profound disease‐associated content and spatial–temporal heterogeneity, engaging in bidirectional crosstalk with pathological progression through interconnected signaling axes. Reciprocally, both pathogenic evolution and therapeutic pressures are confirmed to exacerbate immunosenescence, driving impaired replenishment of immune cells and pathological accumulation of immunosuppressive factors that impact disease progression and poor outcomes. As indicated by clinical evidence, senotherapies designed to eliminate senescent cells or block SASP signaling have emerged as promising interventions to ameliorate age‐related pathologies. In this review, we systematically combed and delineated disease‐specific immunosenescent hallmarks, dissect disease–immunosenescence interplay patterns, and evaluated the translational value of immunosenescence‐targeting strategies.

## Graphical abstract

Along with organismal aging or pathogenic processes especially tumor, multiple immune cell types exhibit those well‐established hallmarks of cellular senescent phenotype, mainly characterized by irreversible cell cycle arrest, metabolic disorder, and over production of SASP. They also exhibit profound phenotypic and functional dysregulation, such as diminished cytotoxicity and upregulated immune inhibitory properties, leading to systematic functional deterioration of immune system.

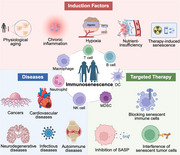

## 1 Introduction

For centuries, it has been widely observed that the elderly exhibit a higher incidence of infectious diseases, cancers, autoimmune disorders, and chronic inflammatory conditions compared with younger individuals. Furthermore, older adults experience more severe disease progression and higher mortality rates following infections. Autopsy and histological studies have revealed significant atrophy and structural alterations in lymphoid tissues (e.g., thymus, lymph nodes, spleen) in aged individuals, suggesting an association with diminished systemic immune responsiveness [1].

The concept of immunosenescence was first proposed by Roy Walford in 1969, who posited that beyond cellular and organ‐level aging, immune system undergoes an age‐related, systemic functional decline [2, 3]. On the one hand, along with organismal aging, multiple immune cell types exhibit those well‐established hallmarks of cellular senescent phenotype, mainly characterized by irreversible cell cycle arrest. Other canonical hallmarks also acquired in senescent cells, such as p16/p21 activation, mitochondrial dysfunction, and overproduction of senescence‐associated secretory phenotype (SASP). On the other hand, systematic functional deterioration of immune system drives evolutionarily maladaptive states during aging or pathological processes. For example, thymic involution depletes output of naïve T cells and myeloid‐biased hematopoiesis. Concurrently, immune cells exhibit profound phenotypic and functional dysregulation, such as diminished cytotoxicity, upregulated inhibitory receptors (e.g., programmed death 1 [PD‐1]; and T cell immunoglobulin [Ig] domain and mucin domain 3 [Tim‐3]), and contracted antigen receptor diversity [2]. These immunosenescence‐associated alterations undermine pathogen defense and clearance capacity toward abnormal cell.

Since the late 20th century, molecular drivers and functional consequences of immunosenescence have been progressively explored. Diverse environmental stressors and cellular elements (e.g., oxidative stress, oncogene activation, and SASP) have been demonstrated to participated in propelling cellular senescence. In 2000, Franceschi et al. identified “inflammaging” as a hallmark of immunosenescence: namely, a chronic, low‐grade, systemic inflammatory state in the elderly population, characterized by persistently elevated levels of multiple proinflammatory cytokines (e.g., interleukin‐6 [IL]‐6; IL‐1β; tumor necrosis factor‐α [TNF‐α]; and C‐reactive protein [CRP]) [4]. Accumulating evidence establishes immunosenescence as a multidimensional, dynamically reprogrammed state across different pathological contexts. These factors sustain immune cell hyperactivation, thereby expanding senescent immune cell pools and constrain systematic immunity during disease progression. These hyperactivated proinflammatory factors also directly generate tissue damage, such as damaging vascular endothelial cells and cardiomyocytes in cardiovascular pathologies.

Compared with the role in non‐neoplastic diseases, immunosenescence exerts more complex and context‐dependent effects on tumorigenesis. Although senescence impairs immune surveillance, significantly increasing the frequency of oncogenic mutations and cancer risk, it fails to explain the paradoxical decline in incidence rates for several cancers in advanced age. Analysis of U.S. cancer statistics reveals a bimodal age distribution for overall cancer incidence and six specific malignancies (e.g., colorectal cancer, leukemia, lung cancer), with incidence peaking in late middle age before declining in geriatric populations. Notably, similar phenomenon has been recapitulated in experimental models, indicating stage‐specific regulatory mechanisms of aging in carcinogenesis [5, 6]. Along with tumor growth, antitumor immunity is attacked and subverted to intensify immunosenescence through competitive sequestration of essential nutrients, induction of chronic inflammation, or strategic engagement of immune checkpoint pathways. Furthermore, cancer cell‐specific SASP factors establish profoundly immunosuppressive niches that promote treatment resistance via T‐cell exhaustion and stromal remodeling.

Although cellular senescence features and molecular mechanisms have been extensively characterized, defining specific hallmarks of immunosenescence remains an ongoing challenge. In this review, we integrate lineage‐specific immunosenescence phenotypes across immune cell types, delineating well‐established characteristics, driving factors, and underlying molecular mechanisms. We further explore immunosenescence signatures in diverse diseases (e.g., degenerative diseases and malignancies), with particular focus on disease‐associated SASP heterogeneity and its bidirectional interplay with pathology. Given the pervasive value of immunosenescence in oncogenesis and treatment resistance, we critically evaluate therapeutic opportunities for targeting senescent immune cells in oncology. These mechanistic insights reveal actionable targets, distinguishing our synthesis from prior reviews through its translational emphasis on immunosenescence‐directed interventions.

## 2 Hallmarks of Senescence and Immunosenescence

**FIGURE 1:**
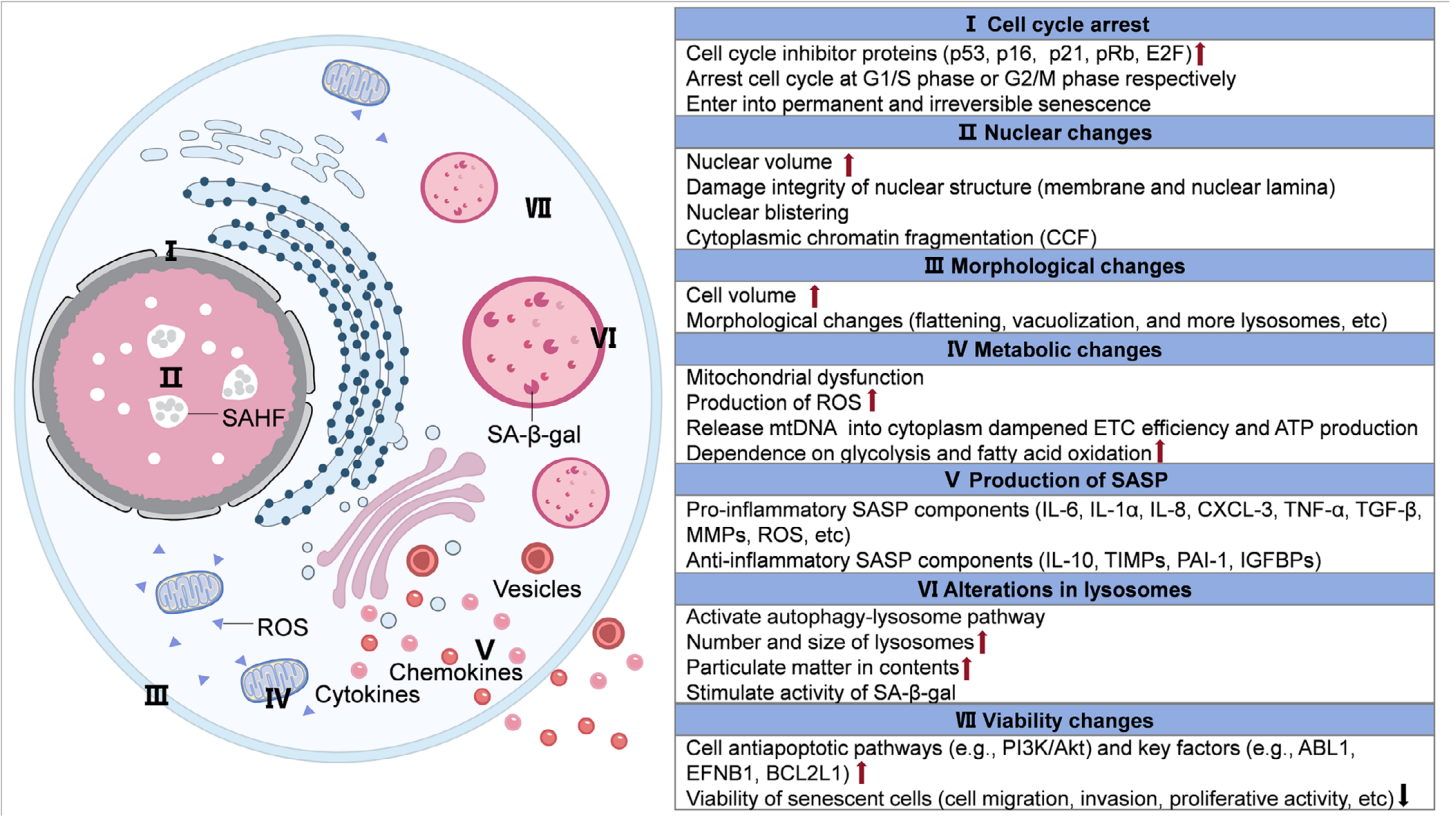
Hallmarks of cellular senescence. This schematic illustrates key morphological and molecular features characteristic of senescent cells. (I) Senescent cells establish irreversible proliferation arrest through sustained activation of p53/p21 and p16^INK4A^/pRb pathways, enforcing permanent cell cycle exit. (II) Nuclear architecture undergoes significant disruption, manifesting as Lamin B1 degradation and nuclear envelope destabilization, formation of senescence‐associated heterochromatin foci (SAHF), cytoplasmic chromatin fragment (CCF) accumulation. (III) Morphological changes include enlarged and flattened cell shape, increased vacuolization, and cytoskeletal reorganization. (IV) Metabolic reprogramming, manifested as mitochondrial dysfunction, elevated ROS production, and increased reliance on glycolysis and fatty acid oxidation. (V) Robust production of senescence‐associated secretory phenotype (SASP) factors such as proinflammatory cytokines chemokines and growth factors. (VI) Marked increase in lysosomal mass, detectable via enhanced senescence‐associated β‐galactosidase (SA‐β‐gal) activity. (VII) Despite proliferative quiescence, senescent cells maintain viability through activation of proliferative pathway (e.g., PI3K–AKT pathway) and upregulation of antiapoptotic proteins (e.g., BCL‐2).

The phenomenon of cellular senescence was firstly described in 1961 [3]. Normal human fetal fibroblasts can maximumly divide about 50 times before entering a senescent state, which is defined with “replicative senescence” or “Hefrederik limit.” Senescence is ubiquitous in various cell types, impacting their biological function and effects toward tumor suppression, embryonic development, wound healing, and other pathogenic processes [4, 5]. Occurrence of cell senescence is generally considered to be a dynamic responsive process of passively accumulated damage. Under accumulated intrinsic damage or continuous stimulation of environmental pressures, senescent cells lose their proliferative ability and fail to enter mitosis by normal physiological stimuli, as well as gradually declined differentiation ability and biological function (Figure 1) [6]. Arrested growth of senescent cells is usually permanent and irreversible. During this process, expression of a number of cell cycle inhibitor proteins increases, especially cyclin‐dependent kinases (CDK) inhibitors p16^INK4A^ (abbreviated as p16) and p21, resulting in arrested cell cycle at G1/S phase or G2/M phase respectively. The former one p16^INK4A^ inactivates CDK4/6 to accumulate phosphorylated retinoblastoma suppressor protein (pRb) and E2 promoter binding factor (E2F) transcription factors, ultimately leading to cell growth arrest at G1/S phase [7]. The latter one p21CIP1/WAF1 blocks CDK1/cycB in a p53‐dependent manner, preventing cell cycle transition from G2 to M phase. Under sustained stimulation of stress such as persistent DNA damage, temporary cell cycle arrest events qualitatively change into permanent and irreversible senescence phenotype. In addition, expression of proteins involved in antiapoptotic pathway (e.g., Abelson murine leukemia viral oncogene homolog 1; ephrin‐B1; B‐cell lymphoma‐2 [Bcl‐2] like 1) is usually upregulated in senescent cells, mediating their resistance to apoptotic signals and cell death [8].

Compared with nonsenescent cells, there are marked morphological changes in senescent cells. Senescent cells are featured with larger size of cell body and more irregular morphology, such as flattening, vacuolization, and abundant lysosomes [9, 10]. Aging‐related cell morphological changes are relied on cytoskeletal rearrangement dominated by vimentin rearrangement, which is associated with abnormally activated transcription factor 6α and nuclear factor kappa‐B (NF‐κB) pathways [11]. Changes in structure and contents of nuclear are also typical features of senescent cells. There is significant amplified size of nuclear, damaged nuclear membrane and nuclear lamina, nuclear blistering, and formation of cytoplasmic chromatin fragmentation (CCF). Changes in composition of the nuclear lamina break anchor of nuclear lamina association domains on nuclear lamina, change original perinuclear localization of heterochromatin and chromatin compartments to alter transcriptional activities of these areas [12]. In particular, frequency of long‐range (>2 Mb) interaction on chromatin is obviously reduced in senescent cells [13].

The metabolic pattern and behaviors are also greatly altered in senescent cell, manifested with mitochondrial dysfunction and anabatic redox activity [8]. The number of mitochondria is accumulated, whereas the permeability of mitochondrial membrane increases to permit release of mitochondrial DNA into cytoplasm [14]. Meanwhile, the efficiency of electron transport chain and adenosine triphosphate (ATP) production are dampened, resulting in decreased mitochondrial membrane potential and increased reactive oxygen species (ROS) production. Under this circumstance, almost senescent cells undergo a switch of metabolic pattern, with increased dependence on glycolysis and fatty acid oxidation, which also increases production of associated metabolites [15]. In particular, increased content of glyceraldehyde‐3‐phosphate and 3‐phosphoglycerate, implied that major intermediate metabolites such as citrate, alpha‐ketoglutarate (α‐KG), and glutamate are furtherly utilized [16]. In addition, cholesterol metabolic pathways are also significantly augmented in senescent cells, with accumulated phospholipids, ceramides, fatty acids, cholesterol, which might impair mitochondrial homeostasis [17, 18]. In order to reassign and recycle those excessive metabolic products, lysosome‐mediated autophagy is significantly activated in senescent cells accordingly. Both number and size of lysosomes are observed to be increased, while accumulation of particle contents inside lysosomes leads to the enlargement of cell body [19]. β‐Galactosidase (β‐gal) is an endogenous lysosomal enzyme encoded by the galactosidase beta 1 gene, which serves to remove galactose residues from various proteins and nonprotein substrates. Markedly enriched β‐gal is regarded as one of the classic senescent biomarkers (namely, senescence‐associated β‐gal, SA‐β‐gal).

Under healthy physiological condition, senescent cells and SASP components can be recognized and removed by intracellular lysosomal network to ensure cellular homeostasis, or be eliminated by triggering circulating immune cells such as natural killer (NK) cells to delay aging process [20, 21]. However, to be noted, emerging senescence in immune cells is inevitable during host aging, leading to decreased activity and dysfunction of both innate and acquired immunity. Besides with aging, systematic immunosenescence is a well‐known risk factor for development of aging‐related diseases and vice versa. For instance, immunosenescence can be present in the early stage of cancer formation and is aggravated accompanied by carcinogenesis. The event of immunosenescence has been observed in patients with colorectal cancer, lung cancer and ovarian cancer, which is majorly characterized by decreased proportion of CD8^+^ T cells [22, 23]. Meanwhile, failure of removing tumor cells or abnormal cells by senescent immune system expands immune fatigue. Moreover, the proinflammatory SASPs components promote tumor invasiveness and other malignant properties [24]. Appearance of immunosenescent signature in patients with tumor is also associated with infiltration of lymphocyte and protumor immune cells (e.g., regulatory T [Treg] cells; and myeloid‐derived suppressor cells [MDSCs]), impacting their responses to immune therapy [25]. Therefore, elucidating the intricate crosstalk between aging‐related diseases and immunosenescence is necessary to recover immune surveillance function.

Recent advances in artificial intelligence (AI) and machine learning (ML) have significantly updated our understanding of dynamic characters and mechanistic interpretation of immunosenescence across diverse disease contexts (Table 1). On the one hand, beyond those established molecular markers, multiomics‐based technology has been applied in identifying novel immunosenescence signatures, spanning epigenetic modifications (e.g., methylation sites), peptide profiles (e.g., urokinase‐type plasminogen activator receptor), and posttranslational modifications, which enables precise discrimination of senescent immune subsets [26]. Through weighted scoring of those core biomarkers (e.g., *cyclin‐dependent kinase inhibitor 2A*, *CDKN2A*; *C‐X‐C motif chemokine ligand 8*, *CXCL8*; *high mobility group box 1*, *HMGB1*; *p16*), quantitative indices of immunosenescence are constructed, such as the ImmunoSen‐Index [27]. On the other hand, multimodal data undergo integration via deep learning architectures (*e.g., Transformers; graph neural networks, GNNs*) to screen senescence‐specific subpopulations at single‐cell resolution. For instance, the *siAge* model constructed the first dynamic atlas of human peripheral immune cells from birth to advanced age (0–90 years), delineating their functional trajectories across developmental stages [28]. Similarly, single‐cell transcriptomic data collected by 1081 healthy individuals is used to establish the *sc‐ImmuAging* clock, revealing infection‐specific immunosenescence heterogeneity [29]. Furthermore, incorporating T‐cell receptor (TCR) and B‐cell receptor repertoire information contribute to clarify the molecular boundaries in distinguishing senescent lymphocytes [30]. Considering the multifaceted interplay between metabolic dysregulation, inflammatory responses, and epigenetic reprogramming in immunosenescence, AI‐driven integration of metabolic clocks and epigenetic clocks further decipher the value of distinct biological reactions in immunosenescence [31, 32]. With the advancement in AI‐based approaches, molecular landscape and secretory profiles of immune aging are illuminated, enabling more precise prediction of senescence trajectories.

**TABLE 1:** Summary of AI/ML‐based immunosenescence models.

Year	Model	Participants	Specimen	Analytes	Regression models	References
2019	IMM‐AGE score	*n*: 135; age: 25–76; population: healthy control	PBMCs	Immune cell subset‐specific gene expression for deriving clinically meaningful immune age metrics	Linear regression, Cox regression	[33]
2020	Lymphocyte subset absolute counts for modeling	*n*: 227; age: 30–84; population: healthy control, NSCLC patients	Peripheral whole blood	Lymphocyte subset absolute counts as independent predictors of progression‐free survival in NSCLC	Binary logistic regression, Cox regression	[34]
2020	Antibody‐bound peptides for modeling	*n*: 1675; population: general population	Plasma	Antibody‐bound peptides for establishing immune age metrics	Elastic net regression, linear regression, machine learning regression	[35]
2021	iAge	*n*: 1001; age: 8–96; population: healthy control, ME patients, CFS patients	PBMCs	Multiomics blood immunome profiles for predicting composite aging phenotypes	Linear regression, multiple regression, LASSO regression, Cox regression	[36]
2022	Integrated transcriptomic signatures for modeling	*n*: 1896; age: 0–≥60; population: AML patients	Plasma	Integrated transcriptomic signatures (IED) for AML risk stratification and immunotherapy response prediction	Cox regression, LASSO‐penalized regression	[37]
2023	High‐dimensional Immunophenotyping for modeling	*n*: 43,096; age: 20–88; population: healthy control	Serum, plasma	High‐dimensional Immunophenotyping (T/NK subsets) for immune‐age prediction	Linear regression, Random Forest regression	[38]
2023	Organ‐derived plasma proteins for modeling	*n*: 5,676; population: healthy control, DAT patients	Plasma	Organ‐derived plasma proteins for quantifying organ‐specific aging and predicting disease risks (11 organs)	LASSO regression, Cox regression, linear regression	[39]
2024	IntrinClock	Combined with data from GSE41826, GSE42861, GSE42861, and so on	PBMCs	Cell‐intrinsic aging signals isolated from CpG methylation, independent of immune cell composition changes	Linear regression	[40]
2024	CS Predicting Model	Combined with data from 33 cancer types in TCGA	Plasma	Pan‐cancer senescence signatures integrating telomere dynamics and immune infiltration for therapy response prediction	Cox regression	[25]
2024	MLIRS score	*n*: 58; population: pancreatic cancer patients; combined with data from TCGA‐PAAD, GSE28735, GSE57495, GSE62452, GSE79668, GSE85916, PACA‐AU‐seq, PACA‐CA‐array, and EMTAB6134	Pancreatic tissues from patients with pancreatic cancer	13 immunosenescence‐regulating markers for prognostic modeling	Cox regression	[41]
2025	Single‐cell RNA and T cell/B cell receptor sequencing for modeling	*n*: 220; age: 0–90+; population: healthy control	PBMCs	Lifecycle‐wide single‐cell profiles for immune age prediction	Linear regression, LOESS regression, Random Forest regression	[28]
2025	sc‐ImmuAging	*n*: 1081; age: 18–97; population: healthy control	PBMCs	Single‐cell transcriptomes for modeling and predicting immune function alterations	LASSO regression, linear regression	[29]
2025	Circulating cytokine levels for modeling	*n*: 72; age (mean): 66; population: iCAD patients, ASCAD patients	Peripheral blood	Immune biomarker signatures distinguishing iCAD (immune activation) from ASCAD (immunosenescence) via CD57 expression and memory subsets for precision management	Random Forest regression	[42]
2025	IC clock	*n*: 1014; age: 20–102; population: healthy control	Serum	DNA methylation IC for predicting composite clinical phenotypes of intrinsic capacity and all‐cause mortality	Continuous two‐phase model regression, elastic net regression, linear regression, logistic regression	[43]
2025	Organ‐derived plasma proteins for modeling	*n*: 44,498; age: 40–70; population: general population	Protein from plasma and CSF	Plasma testing of specific organs for assessing the degree of organ aging and predicting the risk of diseases and mortality rates	LASSO regression, Cox regression, linear regression	[44]
2025	EpInflammAge	*n*: 223; age: 19–101; *n*: 106; age: 25–88; population: healthy control	Plasma	Generating the age estimates and levels of inflammatory parameters for methylation data	mRMR approach; elastic net regression	[45]
2025	Secretome of senescent monocytes for modeling	*n*: >1000; age: 21–102; population: healthy control	Protein from plasma	Monocyte‐derived SASP plasma biomarkers predicting multidimensional aging phenotypes	LASSO regression	[46]

This table display AI/ML‐based immunosenescence models, which are constructed by incorporating multiomics platform data and regression‐based analytical approaches, with applications spanning immune age estimation, disease risk stratification, and therapeutic response prediction. (*n*: number of samples).

*Abbreviations*: AML, acute myeloid leukemia; ASCAD, aortic stenosis (tricuspid valve) and an indication for surgical replacement coronary artery disease; CFS, chronic fatigue syndrome; CS, cellular senescence; CSF, cerebral spinal fluid; CyTOF, cytometry by time of flight; DAT, dementia of the Alzheimer's type; IC, intrinsic capacity; iCAD, isolated coronary artery disease; IED, immune effector dysfunction; ME, myalgic encephalomyelitis; mRMA, minimum redundancy–maximum relevance; NSCLC, non‐small cell lung cancer; PBMCs, peripheral blood mononuclear cells; qRT‐PCR, quantitative reverse transcription polymerase chain reaction; RNA‐Seq, RNA sequencing; SASP, senescence‐associated secretory phenotype; TCGA, the cancer genome atlas.

## 3 Induction Factors of Immunosenescence

Immunosenescence arises through a multifactorial process driven by diverse physiological elements and environmental influences, including intrinsic organismal aging and pathological stress from various diseases. There is also complicated bidirectional relationship between immunosenescence with pathogenesis‐induced tissue damage, as well as diseases‐associated chronic inflammation, hypoxia, and nutritional deficiencies. Furthermore, therapeutic interventions such as radiotherapy and chemotherapy act as potent external stressors, which induce apoptosis and inevitably compromise immune cell functionality, thereby contributing to systemic immune aging and functional decline of immunity (Tables 2 and 3).

**TABLE 2:** Clinical evidence of senescent immune cells in patients.

Diseases	Type of senescent immune cell	Manifestation	References
Multiple myeloma	CD4^+^CD28^−^ T cell	Increased CD4^+^CD28^−^ T cell population	[47]
	T cell	Increased senescent secretory effector phenotype: KLRG‐1^+^/CD57^+^/CD160^+^/CD28^−^	[48]
	CD8^+^ T cell	Decreased CD28 expression; increased CD57 and PD‐1 expression	[49]
Nasopharyngeal carcinoma	CD8^+^CD28^−^ T cell	Increased proportion of CD8^+^CD28^−^ T cell after radiotherapy	[50]
Colorectal cancer	Peripheral immune cell	Decreased p16^INK4A^ expression	[51]
	CD68^+^/CD206^+^ macrophage	Increased infiltration of CD68^+^/CD206^+^ cell	[52]
Breast cancer	T cell	Decreased CD28, CD27, CD86, GRAP, and LRRN3 expression after chemotherapy	[53]
Triple‐negative breast cancer	B cell	Increased CD27^bright^ B cell population	[54]
Non‐small cell lung cancer	T cell	Increased CD57^+^CD28^−^CD8^+^ T cell population after radiotherapy	[55]
	Macrophage	Increased p16^INK4A^ expression	[56]
	Macrophage	Increased protumorigenic SASPs secretion (Bmp2, CCL2, CCL7, CCL8, CCL24, CXCL13, and IL‐10); increased FOLR2 and CD163 secretion	[57]
Prostate cancer	Neutrophil	Increased SA‐β‐Gal secretion	[58]
	M2 Macrophage	Increased CD163 and VSIG4 expression	[59]
	NK cell	Decreased NKG2A expression after surgery	[60]
Cervical cancer	CD8^+^ T cell	Increased CDKN1A expression; decreased CD27 expression after concurrent chemoradiotherapy	[61]
Glioblastoma	GAMs; MDMs	Increased extracranial MDMs in GAMs	[62]
Non‐Hodgkin lymphoma	NK cell	Decreased NKp30, NKp46, and NKG2D expression	[63]
Kaposi sarcoma	NK cell	Decreased NKG2D expression	[64]
Head and neck squamous cell carcinoma	B cell	Increased CD27^bright^B cell population	[65]
	CD8+ T cell	Decreased naive CD28+CD45RO−CD8+ T cell population increased memory CD28+CD45RO+ and effector CD28−CD8+ T cell population	[66]
	Macrophage; CD4^+^ and CD8^+^ lymphocyte	Decreased proportion of CD163^+^ macrophages, CD4^+^ and CD8^+^ lymphocytes	[67]
Gastroesophageal adenocarcinoma	B cell	Increased CD27^bright^B cell population	[65]
Ovarian cancer	T cell	Increased CD28−CD57+CD8+ T cell population; increased SA‐β‐Gal and KLRG‐1 expression; decreased CD27 expression	[68]
Lung cancer Breast cancer melanoma Prostate cancer	T cell	Increased ILT4 expression; increased senescent T cell population	[69]
Rheumatoid arthritis	CD4⁺CD28^−^ T cell	Increased CD4⁺CD28^−^ T cell population in peripheral blood and synovial fluid; increased RANKL expression	[70]
	CD8⁺CD28^−^ T cell	Increased frequency with CD57⁺ phenotype; involving in chronic inflammation; promoting bone loss	[71]
Systemic lupus erythematosus	CD4⁺CD57⁺ T cell	Increased frequency of senescent phenotype cell; increased expression of BCL‐2 and IFN‐stimulated genes	[72]
Multiple sclerosis	CD28^−^ CD8⁺ T cell	Premature senescence of CD8⁺CD28^−^ T cell; impaired proliferative capacity and increased expression of cytotoxic molecules	[73]
Inflammatory bowel disease	Macrophage	Increased p16⁺/p21⁺ intestinal macrophage and senescent CD4⁺ T cell population; increased SASP productive activity, and impaired clearance capacity	[74]
Type 2 diabetes	CD4⁺/CD8⁺ T cell	Increased senescent T cell population in peripheral blood; low‐grade chronic inflammation, cellular stress, altered surface chemokine receptor expression, and impaired glucose uptake	[75]

Manifestation of disease‐specific alterations in senescent immune cell phenotypes and biomarkers across various cancers and multiple diseases.

*Abbreviations*: BCL‐2, B‐cell lymphoma‐2; Bmp2, bone morphogenetic protein 2; CCL, CC chemokine ligand; CDKN1A, cyclin‐dependent kinase inhibitor 1A; CXCL13, C‐X‐C motif chemokine 13; FOLR2, folate receptor 2; GAMs, glioma‐associated macrophages; GRAP, Golgi‐associated Rab acceptor protein; IFN, interferon; IL, interleukin; ILT4, interleukin‐like transcript 4; KLRG‐1, killer cell lectin‐like receptor G1; LRRN3, leucine‐rich repeat neuronal protein 3; MDMs, monocyte‐derived macrophages; NKG2A, natural killer group 2 member A; NKG2D, natural killer group 2 member D; NKp30, nuclear pore protein p30; NKp46, nuclear pore protein p46; PD‐1, programmed death 1; RANKL, receptor activator of nuclear factor‐κB ligand; SASP, senescence‐associated secretory phenotype; SA‐β‐Gal, senescence‐associated β‐galactosidase; VSIG4, V‐set and immunoglobulin domain‐containing 4.

**TABLE 3:** Experimental evidence of senescent immune cells in physiological aging or disease‐associated senescent models.

Model Types	Type of senescent immune cell	Manifestation	References
Physiological aging (mice model)	CD8^+^ T cell; neutrophil	Decreased number of CD8^+^ T cell and neutrophil	[76]
	CD8^+^ T cell	Increased p53 signaling; decreased antitumor responses	[77]
	T cell	Increased group IVA phospholipase A_2_ expression; altered lipid metabolism	[78]
	T cell (blood and spleen)	Decreased CD27 and CD28 expression; increased p53 and p21 protein	[69]
	Macrophage (spleen)	Decreased M1 and M2 markers (IL‐6, IL‐1β, and TNF‐α)	[79]
	NK cell (bone marrow)	Decreased mature NK cell population	[80]
	Thymic precursor lymphocyte	Accumulated DNA damage; undergone cell cycle arrest	[81]
	Ly6C^high^ monocyte (bone marrow and spleen)	Increased Ly6C^high^ monocyte and macrophage population; decreased CSF‐1R expression	[82]
Physiological aging (canine model)	Progenitor B cell	Inhibited proliferation by TNF‐α; induced apoptosis by TNF‐α	[83]
	CD8^+^ T cell	Decreased CD8^+^ T cell proportion	[84]
KRAS‐driven NSCLE (mice model)	Macrophage	Increased p16^INK4A^ expression and SASPs	[57]
Kras‐driven lung cancer (mice model)	Macrophage (via peritoneal lavage)	Increased p16^INK4A^ expression and SASPs; increased CXCR1^High^ macrophage population	[56]
Prostate cancer (mice model)	C_12_FDG^+^ neutrophil	Increased CDKN2A and CDKN1 expression; increased β‐gal activity; increased SASPs secretion	[58]
Melanoma and lung carcinoma (mice model)	T_Tad_ cell (PD‐1^+^Tox^+^IL‐7R^+^CD8^+^ T cell)	Increased proportion of T_Tad_ cell in tumor tissues	[85]
Colorectal cancer (mice model)	Macrophage	Altered TAM expression signature; increased nuclear translocation of p‐NF‐κB in aged mice	[52]
Breast cancer (cell model)	NK cell	Phosphorylated ATM; activated DDR (H2AX, 53BP1, CHK2); increased SA‐β‐Gal^+^ NK cell population	[86]
	T cell	Decreased CD28 expression; shorten telomeres; increased p53/p21/p16 expression	[87]
Autoimmune CNS inflammation (EAE mouse)	Macrophage	Increased Bcl‐2⁺ and p16^INK4A^⁺ senescent myeloid cell population in spinal cord; presenting SASP signature; accumulated with disease	[88]

Experimental evidence of senescent immune cell dysregulation in diverse physiological aging and disease‐related models, detailing specific senescence‐associated phenotype and molecular alterations.

*Abbreviations*: ATM, ataxia telangiectasia mutated; Bcl 2, B‐cell lymphoma‐2; CDKN1, cyclin‐dependent kinase inhibitor 1; CDKN2A, cyclin‐dependent kinase inhibitor 2A; CNS, central nervous system; CSF‐1R, colony stimulating factor 1 receptor; CXCR1, C‐X‐C motif chemokine receptor 1; DDR, DNA damage response; EAE, experimental autoimmune encephalomyelitis; IL, interleukin; NF‐κB, nuclear factor kappa‐B; NSCLE, non‐small cell lung cancer; SASP, senescence‐associated secretory phenotype; SA‐β‐Gal, senescence‐associated β‐galactosidase; TAM, tumor‐associated macrophage; TNF‐α, tumor necrosis factor‐α; TTAD cell, tumor‐infiltrating age‐associated dysfunctional cell; β‐gal, β‐galactosidase.

### 3.1 Physiological Aging

Accompanied with aging of body, immune cells experience inevitable senescence, with both organ reorganization and cellular changes participated in this process [2]. Regenerative function of thymus gradually deteriorates during aging, manifested with expansion of aplastic nonepithelial perivascular space and loss of epithelial space, limiting the number of thymocytes to restrict further development into immune cells especially naive T cells, as well as reducing resistance and immune responses to pathogens [89]. Crucially, served as sources of lymphoid cells (e.g., T cells, B cells, and NK cells) and myeloid cells (e.g., neutrophils, macrophages, etc.), self‐renewal of hematopoietic stem cells (HSCs) is impaired under the inflammation environment of senescent bone marrow. When HSCs derived from aged mice are transplanted into young mice, bone marrow homing and self‐renewal ability of these old HSCs were significantly restricted [90].

As the initiating event of immunosenescence, aging of HSCs is regulated by complicated extracellular signaling molecules. Compared with young HSCs, multiple inflammatory signaling pathways are significantly activated in senescent HSCs, especially manifested with augmented expression of IL‐1β and TNF [91]. Serving as classic immune response regulators, deficiency of Semaphorin4A (Sema4A) leads to enhanced inflammatory sensitivity of HSCs, thereby accelerating aging of immune system [92]. Age‐associated epigenetic alterations constitute a fundamental layer of regulation underlying HSCs senescence. Senescent HSCs often exhibit global DNA hypomethylation alongside the abnormal DNA methylation patterns at specific promoter regions (e.g., tumor suppressor or differentiation‐related genes) [93]. Various epigenetic modification events like histone acetylation, phosphorylation, and heterochromatin accumulation also increase with aging, leading to transcriptional silencing or aberrant activation of genes involved in self‐renewal, differentiation, DNA repair, and inflammatory responses [94]. Along with the development of HSC aging, genetic accessibility of distinct genes switches from closed to open status, such as Clu, Aldh1a1, and Cdc42, while inhibiting these core transcription factors can reset the aging state of HSCs [95].

Under these stimulatory factors, senescent HSCs also gradually show myeloid bias of differentiation, contributing to imbalanced composition of immune cells directly, presented with decreased number of lymphoid cells (e.g., T cells and B cells) but more myeloid cells (e.g., neutrophils and macrophages) [96]. In particular, the ration of naive T cell/memory T cell and diversity of TCR are significantly diminished, while cell population with arrested cell cycle is common among T cell [38, 97, 98]. Compared with healthy HSCs origin, immune cells differentiated from senescent HSCs such as neutrophils and NK cells are generally dysfunctional or fatigue in producing or secreting inflammatory factors. Removement of dysfunctional aging HSCs can correct aging‐related differentiation bias of them, improving the differentiation imbalance of the hematopoietic system in aged mice [99].

### 3.2 Disease‐Associated Aberrant Microenvironmental Stress

#### 3.2.1 Chronic Inflammatory Microenvironment

Inflammation represents complex protective immune response when confronted with infection, injury, or harmful stimuli, aiming to eliminate pathogens, repair tissues, and restore homeostasis. However, chronic inflammatory pressure induced by diverse pathologies propels aging and age‐related diseases through multiple molecular mechanisms, including remodeling the senescence‐associated epigenetic landscape. For example, cyclic GMP–AMP synthase (cGAS)–stimulator of interferon genes (STING)–type I interferon (IFN‐I) cascade, the potent driver axis involved in inflammation and cellular aging, can be activated by depletion of repressive histone marks (e.g., H3K9me3 and H3K27me3) [100, 101]. These aging processes reciprocally exacerbate chronic inflammation and amplify other hallmarks of aging in turn. For instance, systemic or local inflammation induced DNA damage prompts CD8⁺ T cells to enter an aging state, as observed in patients with obesity or chronic obstructive pulmonary disease (COPD) [102, 103]. Similarly, continuous viral antigens exposure (e.g., hepatitis B virus, HBV; and COVID‐19) activates inflammatory signals, which triggers aging phenotypes and impaired functions of T cells [104, 105, 106].

As proved by the cancer immunosurveillance theory, spectrum of inflammatory mediators is initially stimulated by T cells recognized specific tumor antigens, exerting pivotal antitumor role of various aspects, such as IFN‐γ and TNF‐α [107, 108]. Along with tumor development, tumor microenvironment (TME) undergoes evolvement, presented with a subverted and sustained adaptive‐innate immune cell crosstalk. Tumor cells employ sophisticated strategies to subvert immune surveillance, such as release of immunosuppressive mediators (e.g., IL; and angiotensin II [Ang II]) and engagement of immune checkpoint proteins. Concurrently, a cluster of chemokines (e.g., chemokine ligand 22 [CCL22]; and C‐X‐C motif chemokine ligand 12 [CXCL12]) are secreted to promote recruitment and infiltration of immunosuppressive cell populations, creating a permissive environment for tumor proliferation and metastatic dissemination [109].

Within the tumoral chronic inflammatory environment, diverse cytokines (e.g., IL and Ang II) are employed by malignant cells to destroy immune cells by arresting cell cycle or disrupting metabolic processes, ultimately impairing the cytotoxic potential of T cells and other immune effector cells [110, 111, 112]. For example, IL‐2 released by melanoma cells can promote CD8^+^ T cell senescence by activating signal transducer and activator of transcription (STAT)5–5‐hydroxytryptophan–aryl hydrocarbon receptor pathway, labeled with enhanced expression of senescence‐related genes in CD8^+^ T cells (PD‐1), lymphocyte activation gene 3, CD39, Tim‐3, and dysfunctional cytotoxicity (decreased release of IFN‐γ and TNF) [113, 114, 115, 116, 117]. Sustained exposure to interleukins (e.g., IL‐1β, IL‐6, IL‐7) also induces profound mitochondrial dysfunction in CD4^+^ T cells, culminating in cellular senescence by elevating expression of CD57, one of the senescence markers [118, 119, 120]. Apart from interleukin family, senescence of CD4^+^ T cells is under the induction of TNF‐α and IFN‐α, which can be significantly attenuated following pharmacological inhibition of TNF‐α [121]. In breast cancer‐bearing mice models, Ang II derived from breast cancer cells promotes accumulation of C‐X‐C motif chemokine receptor (CXCR)1, which drives expansion of senescent neutrophil population (CXCR4^high^ CD62L^low^) in blood, lung, and liver [122]. Beyond cytokine‐mediated proimmunosenescence effect, direct interactions between T cells and tumor‐expressed immune checkpoint molecules are also confirmed to induce cell cycle arrest and senescence‐associated phenotypes [123]. As is exemplified by in vitro studies, coculture with multiple myeloma cells results in arrested S phase in PD‐1^high^ T cells, accompanied by dysregulation of cell cycle regulators (CDK6 and *E2F3* transcription factors) and enhanced SA‐β‐Gal activity [111].

Updating evidences suggest a more complicated function of tumor‐derived chemokines, such as CCL22 and CXCL12 in orchestrating tumor immune evasion. These chemokines not only recruit diverse immunosuppressive cell populations (e.g., Treg cells and MDSCs), but also exacerbate immune cell senescence and dysfunction of them [124, 125, 126, 127]. Abundant infiltrated Treg cells in TME have been observed in types of tumors, such as ovarian cancer, cervical cancer, breast cancer, which is connected to poorer outcome [128, 129, 130]. Beyond the regular role in cytokine‐mediated immunosuppression, tumor‐infiltrated Treg cells also target on cell cycle dynamics and senescence processes in multiple immune cells. Specifically, Treg cell‐derived transforming growth factor‐β (TGF‐β) and other mediators engage specific surface receptors on dendritic cells (DCs), triggering Janus kinase (JAK)/STAT signaling cascade and subsequent programmed cell death ligand 1 (PD‐L1)–STAT3 signaling to drive senescence of DC ultimately [131]. Furthermore, through depleting of glucose availability in CD8^+^ T cell, Treg cells activate the ataxia telangiectasia‐mutated gene (ATM)‐associated DNA damage response (DDR) pathway to promote cellular senescence and functional impairment of them [132]. Besides, cytokines such as CCL2 and IL‐8 facilitate the recruitment and tumor infiltration of MDSCs, which facilitates senescence of immune cells by both the manner of direct cell‐cell interactions (via PD‐L1/PD‐1 axis) or long‐range communication (through exosomes or soluble factors like TGF‐β) [133, 134, 135]. In colon cancer, MDSCs exploit the PD‐L1/PD‐1 axis to downregulate NK cell‐activating receptors nuclear pore protein p46 (NKp46) and NK group 2 member D (NKG2D), effectively impairing NK cell function and promoting senescence [136]. Additionally, MDSC‐derived membrane‐bound TGF‐β further suppresses NK cell cytotoxicity by reducing surface expression of NKG2D and diminishing IFN‐γ production [137].

#### 3.2.2 Hypoxic Microenvironment

Hypoxia represents a symbolic event in almost solid tumors, originated from their urgent demand for oxygen and nutrient resources, even with the support of tumor neovascular or vascular mimicry. This disparity between oxygen demand and supply generates intratumoral hypoxic regions, which is characterized with abnormal accumulated lactate and hypoxia‐inducible factor‐1α (HIF‐1α), and impaired clearance of intracellular ROS. The hypoxic situation drives metabolic reprogramming in tumor cells, predisposing them into anaerobic glycolysis‐preferred energy production pattern even being placed in normoxic environments, which is defined as the Warburg effect [138]. Although with relatively low energy‐conversion efficiency, Warburg effect of tumor cells endows them with rapid generation of ATP and synthesis of intermediates, to meet the demands of tumor cell proliferation [139]. Along with that, abundant lactate is substantially accumulated to acidize TME, impairing oxygen utilization and exacerbating hypoxic conditions in feedbacks [140, 141].

Lactate, a crucial product of tumor glycolysis, serves as a carbon source to be converted to pyruvate by lactate dehydrogenase to support tricarboxylic acid cycle and proliferation of tumor cells. Clinical and experimental evidence demonstrates high levels of lactate in TME is positively correlated with malignancy and incidence of metastases [142]. Lactate also acts as a potent immunomodulatory signaling molecule, acidifying surrounding TME and eliciting proinflammatory effect [143]. As suggested in renal cell carcinoma, lactate‐rich environment dampened cytokine secretion ability of cytotoxic T lymphocytes (CTLs), through inhibiting phosphorylation of mitogen‐activated protein kinase (MAPK) pathway (p38, c‐Jun N‐terminal kinase [JNK], and c‐Jun), ultimately compromising proliferation and activation of CTLs [144]. Lactate‐induced acidosis activates cyclic adenosine monophosphate (cAMP)‐dependent signals and transfer cAMP into T cells through gap junctions, culminating in DDR activation and T cell senescence [145]. On this basis, IFN‐γ produced by CTLs is heavily blocked by lactate, which potently inhibits NK cell function [146]. The immunosuppressive effect lactate can also be elicited by acidifying intracellular environment, inducing mitochondrial dysfunction and ROS accumulation in immune cells. For instance, in liver metastases of colorectal cancer, elevated lactate levels induce mitochondrial stress in liver‐resident NK cells, impairing their tumoricidal capacity and accelerating NK cell senescence [147]. Furthermore, lactate absorbed by B cells triggers cellular senescence, characterized with increased secretion of SASP factors [148].

In response to hypoxic condition, tumor cells activate HIF‐1α to orchestrate metabolic reprogramming and stimulate angiogenesis. This adaptive response not only enhances anaerobic glycolysis but also boosts excessive secretion of angiogenic factors, resulting in formation of structurally and functionally abnormal vasculature that paradoxically exacerbates tumor hypoxia. Disrupted metabolic processes and activated HIF‐1α signaling pathways within immune cells accelerate cellular senescence and compromise their antitumor immunity. In breast and bladder cancer models, HIF‐1α‐mediated activation of NF‐κB in macrophages upregulates CD47 expression, which in turn promotes macrophage senescence by upregulating senescence‐associated markers p16, p53, and p21 [149, 150, 151]. HIF‐1α also increases quantity of Treg cell, expedites secretion of CTL‐associated antigen‐4 (CTLA‐4) and IL‐10, consequently aborts effector T cell (Teff) from activation and induces senescence [152, 153, 154]. Moreover, HIF‐1α establishes a self‐reinforcing signaling loop via miR‐125a‐5p‐mediated positive feedback pattern, activating NF‐κB pathway to drive secretion of SASP factors, such as IL‐6 and TNF‐α [154].

Hypoxia in TME compromises antioxidant defenses, reducing ROS clearance and creating a redox imbalance. This oxidative stress acts as both an initiator and effector of immune senescence via mitochondrial dysfunction and cell cycle disruption [155]. In melanoma models, ROS directly disrupts immune cell mitochondrial membranes, triggering senescence pathways like mammalian target of rapamycin (mTOR), p53/p21, and NF‐κB [156]. Moreover, ROS stimulates the secretion of SASP components, including matrix metalloproteinase (MMP)‐1, from immune cells. Correspondingly, attenuating ROS levels by ROS scavengers (e.g., N‐acetylcysteine and glutathione) restores proliferative potential and reverses senescence in Treg cells [157].

#### 3.2.3 Nutrient‐Insufficient Microenvironment

Age‐related mitochondrial dysfunction and metabolism disorder have been confirmed in various cells and tissues, serving as an accelerator for the aging of multiple immune cell subtypes [158]. In particular, tumor cells undergo profound metabolic remodeling to accommodate their heightened proliferative demands, which limits the availability of nutrients and exacerbates acidosis. Competing with tumor cells for critical nutrients (e.g., glucose) intensifies the physiological stress of T cells, activating ATM‐related DDR and MAPK‐related pathways to drive cell cycle arrest and senescence [132]. Within this hostile metabolic environment, immune cells in TME undergo extensive metabolic reprogramming, which impairs their survival and function [159].

Anaerobic glycolysis‐dependent catabolism pattern of tumor cells aggravates consumption of nutrients (e.g., glucose, glutamine, arginine) in TME [140, 160]. In particular, restricted glucose directly hinders proliferation, metabolism and function of immune cells, especially T cells [161]. In contrast, exogenously supplementation with glucose restores glucose‐deprived CD8^+^ T cell proliferation, cytokine production, and metabolic function [162]. In CD4^+^ T cells, depriving glucose directly promotes phosphorylation of MAPK and p38, leading to cell cycle arrest and proliferative impairment [163]. As further revealed by in vitro study, being placed in glucose‐restricted conditions impedes glutamine uptake a critical substrate for mitochondrial respiration while disordered mitochondrial metabolism stimulates senescence related signaling pathways (e.g., p16^INK4A^/pRb) in CD4^+^ T cells [164]. Treatment with 2‐deoxy‐d‐glucose, an inhibitor of glycolysis, also induces G1/S phase arrest accompanied by increased senescence of CD8^+^ T cells [165, 166]. Analogously, high consumption of glucose in natural regulatory T (nTreg) cells or tumor‐associated γδTreg cells leads to intratumoral glucose scarcity [132]. Inhibiting glucose transporters and glycolysis in Treg cells attenuates their suppression on Teff cells proliferation, as well as Treg cells‐induced SA‐β‐gal^+^ senescent T cells [132]. In addition to glucose depletion, polyamine metabolic pathways are highly active in tumor cells such as liver cancer cells significantly enhancing the synthesis and excretion of N1‐acetylspermidine. Excreted N1‐acetylspermidine activates SRC signaling pathway in macrophages, driving the polarization toward CCL1^+^ macrophages with immunosuppressive properties [167].

Pathological metabolic dysregulation perturbs core metabolites and metabolic enzymes, thereby profoundly reshape the epigenetic landscape in immune cells especially via those pivotal epigenetic switches, such as DNA methylation and histone modifications. In elderly individuals, CD4⁺ T cells exhibit reduced mitochondrial function and one‐carbon metabolism compared to younger counterparts, a defect mediated by impaired methylation of mitochondrial transfer RNA that compromises energy metabolism [168, 169]. Pervasive mitochondrial dysfunction depletes α‐KG in senescent T cells, inhibiting tet methylcytosine dioxygenase 2 (TET2)‐mediated DNA demethylation and culminating in epigenetic silencing of CD28 and CD27 genes [121]. Furthermore, deficiencies in glucose‐metabolizing enzyme, particularly those governing the nonoxidative pentose phosphate pathway and transketolase, elevate oxidative stress and exacerbate mitochondrial dysfunction, contributing to DNA hypermethylation and functional gene suppression during progressive immunosenescence [170, 171].

### 3.3 Therapy‐Induced Immunosenescence

In addition to immunosenescence caused by intrinsic genetic factors and the aforementioned mechanisms, clinical interventions (e.g., chemotherapy, radiotherapy, and immunosuppressant therapy) represent external factors in inducing immunosenescence. The phenomenon, termed therapy‐induced senescence (TIS), encompasses both direct and indirect pathways leading to immune system senescence and functional decline. Generally, radiotherapy induces apoptosis‐relied cell death in most tumor cells, while a subset of tumor cells enters a senescence state. Meanwhile, both high energy radiation and chemotherapeutic drugs (e.g., etoposide and cisplatin) are prone to generate indiscriminate damage to DNA in immune cells, initiating DDR and following signals to induce senescence [172, 173]. Experimentally simulating therapeutic stressors (e.g., doxorubicin [DOX]; etoposide, VP‐16; γ‐irradiation) also induce telomere shortening, telomerase suppression, and telomeric dysfunction in human T cells [174]. Beyond malignancies, therapeutic regimens for non‐neoplastic conditions (e.g., rheumatoid arthritis [RA] and human immunodeficiency virus [HIV] infection) have also been implicated in driving immunosenescence through direct perturbation of stability of DNA and cell cycle in immune cells. Pharmacologic interventions, including immunosuppressants (e.g., cyclophosphamide), JAK/STAT pathway inhibitors (e.g., tofacitinib), BRAF^V600E^ inhibitor (e.g., vemurafenib), and long‐term antiretroviral therapy, have been shown to induce telomere attrition, sustained activation of ATM/ATR signaling cascades, and upregulation of p16^INK4A^ and p21^CIP1^ in CD4⁺ and CD8⁺ T cells, collectively reflecting persistent engagement of DDR [175, 176, 177, 178]. Therefore, therapy induced cellular senescence and death directly contribute to immunosenescence by destroying quantity, quality, and function of immune cells [179, 180].

Aside from direct cytotoxic effects on immune cells, TIS also generates impaired replenishment of nascent immune cells. Concurrent with impairing circulating immune cells directly, differentiation from HSCs to immune cells is commonly disrupted during treatment with radiotherapy or chemotherapy. Radiotherapy exposure markedly boosts the expression of senescence markers (e.g., SA‐β‐gal and p16^INK4A^/p19^ARF^) of bone marrow stromal cells in mouse models. Subsequently, those senescent stromal cells, coupled with the destroyed bone marrow niche, fail to support generation of HSCs, and even exert deleterious paracrine effects on neighboring cells by secreting SASP especially inflammatory mediators (e.g., ROS) [179, 181]. Chemotherapy drugs (e.g., etoposide) also bring side effects of myelosuppression to induce immune senescence [182].

Beyond hindering immune cell differentiation at its source, a unique cytokine profile‐formed senescent microenvironment is also constructed along with therapy. Under chemoradiotherapy, cytokines like TGF‐β, IL‐1β are released from tumor cells, which can be internalized by immune cells to reprogram cellular metabolism and trigger senescence. Through in‐depth single‐cell sequencing, a specific chemoradiation‐resistant population of cervical cancer cells has been identified, which exhibits robust production of TGF‐β to accelerate senescence of CD8^+^ T cells [61]. Additionally, chemotherapy triggers phosphorylation of NF‐κB signal, leading to release of proinflammatory SASP components in nasopharyngeal carcinoma cells (e.g., IL‐1β, IL‐8, and CCL20) [183]. In patients receiving long‐term hemodialysis, a persistent state of low‐grade systemic inflammation can be induced due to repeated mechanical shear stress, incomplete biocompatibility between dialysis membranes and infusates, and continuous exposure of blood to biomaterial interface. This therapy‐induced inflammatory milieu is marked with chronically elevated circulating levels of proinflammatory cytokines especially IL‐6 and TNF‐α. These factors promote oxidative stress, mitochondrial dysfunction, and sustained activation of DDR in both CD4⁺ and CD8⁺ T cells, ultimately driving their functional exhaustion and immunosenescence [184, 185].

## 4 Manifestation of Senescent Immune Cells and SASP During Immunosenescence

**FIGURE 2:**
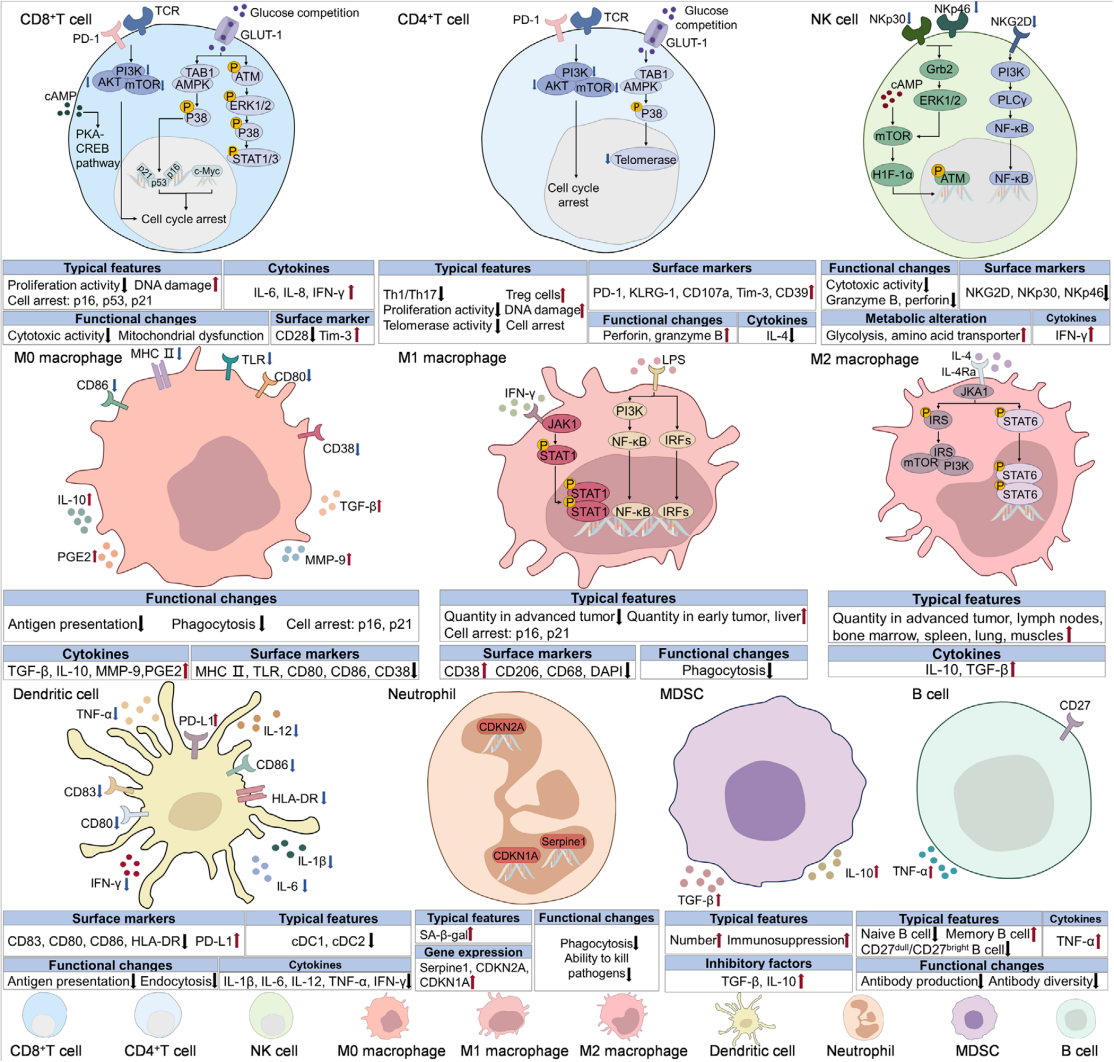
Manifestation of immunosenescence in TME. Tumor microenvironment (TME) drives profound senescence‐related alterations in immune cells, impairing their function and promoting tumor immune evasion. CD8^+^ T cells undergo senescence via PKA–CREB, PI3K–AKT‐mTOR, and p38^MAPK^ pathways, leading to reduced proliferative capacity and cytotoxicity, accumulated DNA damage, increased secretion of IL‐6, IL‐8, and IFN‐γ, downregulation of CD28 and upregulation of Tim‐3, and mitochondrial dysfunction. CD4^+^ T cells are similarly affected by PI3K–AKT–mTOR and p38 signaling, resulting in decreased Th1/Th17 ratio and proliferation, expanded Treg cell populations, reduced telomerase activity, upregulated PD‐1 and other inhibitory receptors, and altered cytokine profiles. Senescent M0 macrophages exhibit impaired phagocytosis, elevated p16 and p21 expression, reduced antigen presentation but increased TGF‐β and IL‐10 secretion. Senescent M1 and M2 macrophages present altered makers respectively (M1: upregulated CD38, p16, p21, downregulated CD206/CD63. M2: upregulated TGF‐β and IL‐10). Senescence of NK Cell is regulated by ERK1/2 and PI3K pathways, leading to impaired cytotoxicity and glycolysis, reduced GZMB/perforin release, and expression of activating receptors (NKp30, NKp46, NKG2D). Senescent B cells display depleted naïve populations, manifested as altered CD27^dull^/CD27^bright^ ratios, reduced antibody diversity but increased TNF‐α secretion. Other senescent myeloid populations like neutrophils exhibit limited phagocytosis to expand malignancy.

During the process of immunosenescence, diverse immune cells including NK cells, macrophages, B cells, and T cells exhibit core shared hallmarks, such as accrued genomic damage and intracellular metabolic dysregulation. Concurrently, distinct phenotypic alterations and multidimensional functional exhaustion manifest across cell types, characterized by impaired cytotoxicity, diminished phagocytic capacity, and attenuated plasticity of adaptive responses. SASP further remodels inflammatory and metabolic landscapes, collectively shaping disease‐associated heterogeneous immune microenvironments (Figure 2). These aging‐associated immunological features drive systemic immune remodeling, thereby increasing susceptibility to infections, malignancies, and chronic inflammatory pathologies.

### 4.1 Typical Features of Senescent Immune Cells

#### 4.1.1 NK Cell

NK cells serve as critical effectors of innate immunity, which are responsible for recognizing and eliminating aberrant cells, such as senescent or tumor cells. Their activity is governed by both surface activating and inhibitory receptors, such as CD16 and CD56, as well as immune checkpoint molecules. Based on the expression of CD16 and CD56, NK cells are conventionally categorized into two functional distinct subsets: immature (CD16^+^CD56^dim^) and mature subtypes (CD16^+^CD56^bright^). CD56^bright^ NK cells primarily secrete cytokines such as IFN‐γ and TNF‐α to act immunomodulatory role. In contrast, the CD56^dim^ subset constitutes the predominant subgroup in peripheral blood, eliminating tumor cells at immune synapses and secreting cytotoxic molecules, such as perforin and granzyme.

**FIGURE 3:**
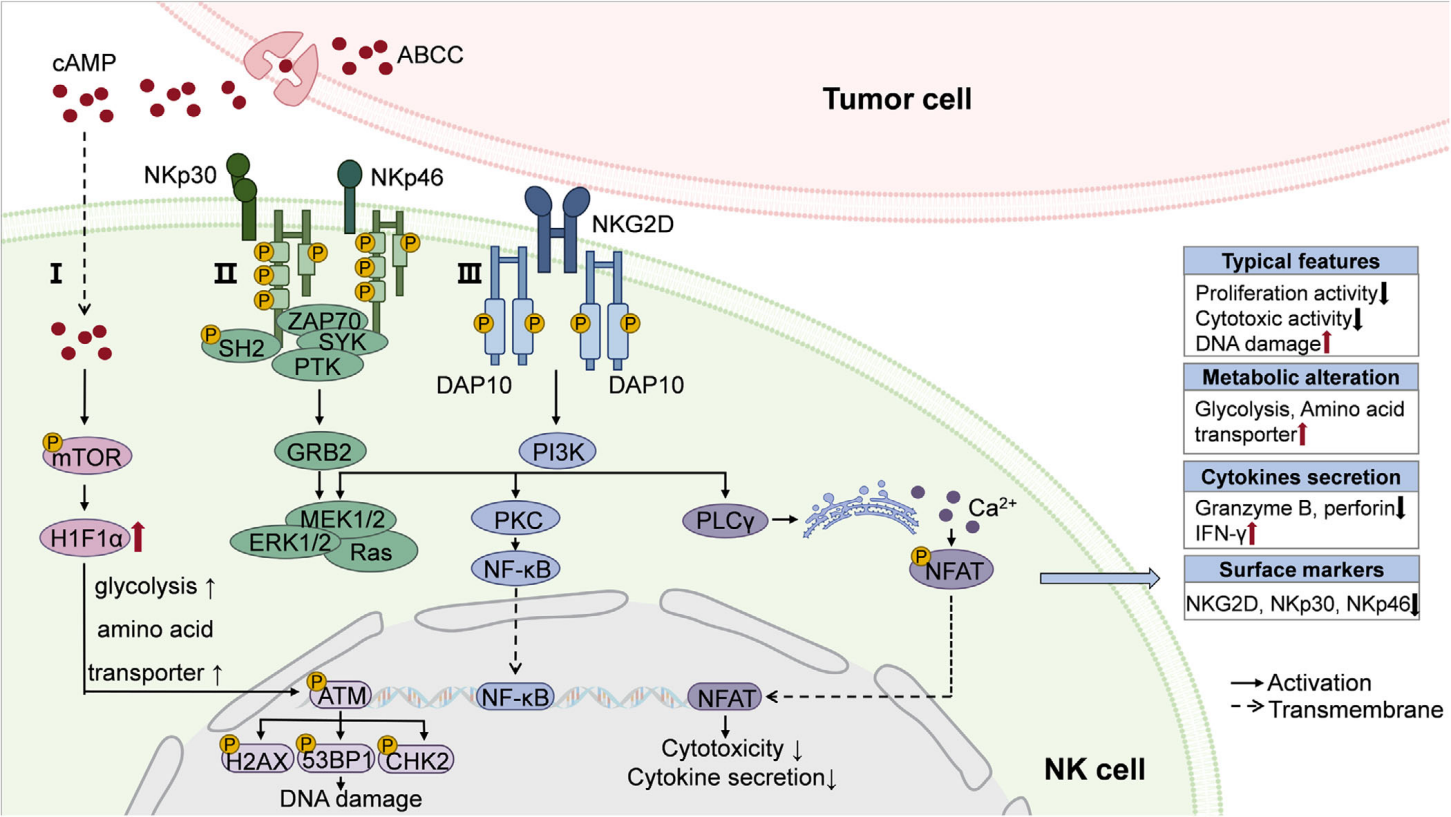
Molecular pathways involved in tumor‐associated NK cell senescence. Characterized by downregulation of activating receptors (NKp30, NKp46, NKG2D) and functional impairment, tumor drives NK cell senescence through distinct molecular pathways: (I) Metabolic reprogramming and DNA damage response. (II) Downregulated NKp30/NKp46 signaling axis. (III) Failure of ligand‐engaged NKG2D in inducing DAP10 phosphorylation and subsequent PI3K pathway activation, leading to reduced secretion of IFN‐γ, TNF‐α, and IL‐2 and inhibited cytotoxic activity.

Organismal aging profoundly alters NK cell biology, manifested with skewed subset distribution (e.g., declined CD56^bright^/CD56^dim^ ratio), phenotypic alterations (e.g., reduced expression of activating receptors like nuclear pore protein p30, NKp30), expansion of CD56^dim^CD57^+^ NK cells, reduced cytokine secretion, and cytotoxicity against target cells (Figure 3) [186, 187]. In particular, NK cells with CD56^dim^CD57^+^ are described as a reliable symbol of senescent NK cells, which has been detected in patients with atherosclerosis, Parkinson disease (PD), or underwent human cytomegalovirus infection [188, 189, 190]. These age‐related changes are conserved in mice, with significant reductions in mature NK cell populations in lymphoid organs of aged animals [80]. In patients with Alzheimer's disease (AD), the number of NK cells also significantly decreases [191]. The altered subset composition in elderly individuals correlates with diminished TNF‐α and IFN‐γ secretion capacity, which persists even following IL‐2 or IL‐12 stimulation [192]. Furthermore, in vitro studies demonstrate that aging compromises the cytotoxic machinery of NK cells. In a series of NK cell–leukemia cell coculture experiments, perforin produced by NK cells from elderly donors are reduced compared with those from younger counterparts. This perforin deficiency directly contributes to the age‐associated decline in NK‐mediated tumor cell killing, representing a key mechanism of immune senescence in the NK compartment [193].

In addition to subset redistribution, senescent NK cells exhibit unique characteristic phenotypics. A hallmark feature of NK cell senescence is the downregulation of surface activating receptors, particularly natural cytotoxicity receptors (NCRs; including NKp30 and NKp46) and NKG2 family members, which significantly compromises their antigen recognition capacity. This receptor deficiency leads to impaired cytokine production and diminished cytotoxic function against target cells. Mechanistically, these critical receptors mediate NK cell activation through distinct ligand interactions: NKG2D (a C‐type lectin‐like receptor) recognizes major histocompatibility complex class I‐related molecules expressed on tumor cells, while NCRs (NKp30 and NKp46) bind to immunoreceptor tyrosine‐based activation motif (ITAM)‐bearing ligands [194]. Accumulating evidence indicates that tumor‐derived factors downregulate NKp30, NKp46, and NKG2D expression, resulting in diminished cytotoxic activity of NK cells against tumors and contributing to NK cell senescence. Clinical observations consistently demonstrate this phenomenon across various malignancies. In patients with non‐Hodgkin lymphoma or acute myeloid leukemia, significant downregulation of NKp30, NKp46, and NKG2D has been well documented [195, 196]. Similarly, Kaposi's sarcoma patients exhibit markedly reduced expression of NKG2D and NKp30 compared with healthy controls, with receptor levels being restored following successful treatment‐induced remission [64].

Senescence of NK cell also performed with metabolic reprogramming, especially alterations in glucose and lipid metabolism mediated by tumor‐derived cAMP [197, 198]. Collected from murine breast cancer models or when cocultured with breast cancer cells, uptake of tumor‐derived cAMP markedly increased SA‐β‐Gal^+^ NK cells, along with impaired expression of effector molecules like granzymes A/B (GZMA/B), perforin, and IFN‐γ. Through high‐throughput sequencing, elevated transcriptional activity of metabolism‐associated genes flux and glucose/lipid metabolic level are unearthed in those senescent NK cells. Besides, treatment of cAMP inhibitor substantially attenuated breast cancer cell‐induced senescence in both murine and human NK cells. Parallel phenomena were observed in melanoma models. For instance, tumor‐derived cAMP in melanoma‐bearing mice upregulated glycolipid metabolism‐related genes in murine NK cells, which is repeatable in human NK cells cocultured with melanoma cell, accompanied with elevated senescent SA‐β‐Gal^+^ NK cells [86].

#### 4.1.2 Macrophage

Macrophages are regarded as primary effectors of adaptive immunity, orchestrating the recognition, phagocytosis, and degradation of cellular debris and pathogens through intracellular degradation mechanisms including autophagy. Senescent macrophages exhibit sustained metabolic impairment that alters polarization dynamics and drives functional decline, manifesting as diminished antigen presentation and loss of phagocytic capacity. Macrophages acquire senescent characteristics during both physiological aging and pathological conditions, exhibiting imbalanced polarization states and elevated expression of senescence markers (p16^INK4A^, SA‐β‐gal). As for macrophages in aged murine models, there is tissue‐specific polarization patterns. For instance, immunosuppressive M2 macrophages are observed predominantly accumulated in bone marrow, spleen, lymph nodes, lungs, and muscle tissues of aged mice [199, 200]. In comparably, proinflammatory M1 macrophages are enriched in adipose tissue and liver [201, 202]. Except for natural aging processes, p16^INK4A^‐positive senescent macrophage populations expand under diverse pathogenic conditions, including PD, osteoarthritis, streptozotocin‐induced diabetes, and ionizing radiation exposure [203, 204]. Radiation‐induced senescence specifically increases SA‐β‐gal^+^ bone marrow‐derived monocytes/macrophages [205].

Tumor‐associated macrophages (TAMs) represent a critical cellular constituent of TME, constituting one of the most prevalent immune cell populations across diverse malignancies [206]. During initial tumor development, malignant cells recruit macrophages through paracrine secretion of cytokines and chemokines, including granulocyte–macrophage colony‐stimulating factor (GM‐CSF), CCL2, and CXCL4. TAMs polarization exhibits microenvironment‐dependent plasticity, with functional specialization dictated by local signaling milieus. Proinflammatory M1‐like TAMs emerge in response to GM‐CSF and IFN‐γ stimulation, characterized by STAT1 and suppressor of cytokine signaling three pathway activation, which promotes antitumor immunity [207]. Conversely, exposure to colony‐stimulating factor 1 (CSF‐1), IL‐4, and IL‐13 induces M2‐like polarization, associated with immunosuppressive and tumor‐promoting functions [208]. This dynamic polarization capacity enables TAMs to perform content‐dependent roles throughout tumor progression and in response to therapeutic interventions.

When being confronted with tumor, senescent signatures always endow TAM populations with discordant polarization and immunosuppressive capacity to assistant tumor progression, as evidenced by clinical observations. For example, markers of M2 macrophage (e.g., CD163; V set and Ig domain‐containing 4, VSIG4) are upregulated pronouncedly in elderly (>60 years) or prostate cancer patients [59]. This phenomenon is recapitulated in KRAS‐driven lung cancer models, where CD206^+^ M2‐polarized TAMs coexpress the senescence marker p16 [57]. In accompany with those TAMs, a subset of tissue‐resident alveolar macrophages (AMs) also exhibit senescence‐associated signatures, including elevated expression of cell cycle inhibitory gene (e.g., Ctnnb1), suggesting an indirect tumor‐mediated induction of macrophage senescence [56]. Similar evidence has been collected in glioblastoma models, where tumor‐derived IL‐6 activates STAT3 signaling to initiate senescence in peritumoral macrophages rather than TAMs [209]. The presence of senescent TAMs has been confirmed to impair antitumor immunity by suppressing infiltration and function of CD8^+^ T cells. Senescent AMs in lung cancer models restrict accumulation of CD8^+^ T cells, while depletion of AM successfully restores infiltration of CD8^+^ T cells [57]. Similarly, senescent macrophages in glioblastoma attenuate response of T cells by producing arginase‐1 (Arg1) to downregulate expression of CD3ζ in T cells [209].

#### 4.1.3 B Cell

As an essential component of adaptive immune system, B lymphocytes undergo a tightly regulated differentiation process beginning with pro‐B and pre‐B cell stages before maturing into naive B cells characterized by high IgD and low IgM expression. Upon tumor antigen encounter, naive B cells experience clonal expansion and differentiate into matured plasma cells or memory B cells respectively. Specific antibodies are synthesized and secreted by matured plasma cells to facilitate effector cells recruitment (e.g., NK cells, macrophages) and mediate their antibody‐dependent cytotoxic effect [210]. Memory B cells can be divided into CD27^dull^ and CD27^bright^ subpopulations, while the former one promptly differentiate into plasma cells when recognizing tumor cells. Comparably, upon antigen re‐exposure, CD27^bright^ B cells can also be differentiated into plasma cells and secreted specific antibodies toward tumor cells in a relatively tardive mode [211].

During the process of aging, there are significant alterations in both quantity and function of B lymphocytes, manifesting as decreased pro‐B cell numbers alongside expanded memory B cell compartments. Major changes of this age‐associated lymphoid‐myeloid shift include reduced CD27^dull^/CD27^bright^ B cell ratio, and diminished antibody repertoire diversity. The myeloid‐biased differentiation of senescent HSCs is responsible for dampened production of pro‐B cells [95]. TNF‐α secreted by aged B cells suppresses proliferation and induces programmed cell death of pro‐B cells [212]. The nuclear structure of aging pro‐B cells also experiences architectural changes, reduced topological domain on Ig heavy chain (IgH) sites related to B cell development hindered the differentiation of progenitor B cells and caused cell apoptosis [213]. Similar phenomenon has also been observed in aged canine models [83]. In comparison, the number of memory B cells exhibits age‐related expansion, and the ratio of CD27^dull^ /CD27^bright^ B cells in memory B cells decreases during aging [214]. Comparative analyses reveal significant reduction in CD27^dull^ subsets with concomitant dominance of CD27^bright^ populations in elderly individuals, which is a phenotypic shift associated with impaired neoantigen responsiveness [211, 215].

Furthermore, senescent B cells display marked reduction in both antibody repertoire diversity and production capacity [216, 217]. Experimental evidence from in vitro stimulation assays demonstrates that IgM and IgA in peripheral blood B cells from the elderly are diminished compared with younger counterparts [215]. This phenomenon is also observed in lymphoid follicles of ileum [217]. The transcription factor E47 in senescent B cells downregulates activation‐induced cytidine deaminase (AID), failing to catalyzing class switch recombination and somatic hypermutation (SHM), blocking conversion from IgM, IgD to distinct secondary isotype antibodies (IgG, IgA, IgE), ultimately reducing antibody diversity and synthesis [218, 219].

Pathological conditions such as chronic infections or malignancies are confirmed to induce sustained inflammation and senescence in B cells. For instance, in the circumstances of autoimmune diseases (systemic lupus erythematosus [SLE] and RA) or AD, B cells are driven to differentiate into age‐related phenotype by abnormally accumulated proinflammatory factors such as IL‐6 and TNF‐α [220, 221]. Senescence of B cells appears after being infected with HIV even in young individuals [222]. Similarly, chronic inflammatory environment of tumor is also found to drive senescence of B cells. As exemplified in triple‐negative breast cancer (TNBC), breast cancer cells provoke myeloid‐biased niche competition between pro‐B cells and myelocytes in bone marrow, ultimately leading to pro‐B cell depletion [223]. Furthermore, increased proportion of memory B cell has been documented across multiple malignancies, including breast carcinoma, non‐small cell lung cancer (NSCLC), and ovarian cancer [224, 225, 226]. As for B lymphocytes infiltrated inside tumor‐associated tertiary lymphoid structures (TLS), CD27^dull^/CD27^bright^ B cell ratios demonstrate significant reduction, which is correlated with unfavorable clinical outcomes in head and neck squamous cell carcinoma (HNSCC) and TNBC [54, 65]. B cells‐related immunosenescence is also marked with impaired humoral immunity, featured with antibody diversity restriction and localized antibody deficiency. For example, in cutaneous melanoma, higher diversity of antibodies production is associated with higher proportion of B cells expressing AID and performing SHM, which is positively related to improved prognosis [227]. Compared with peripheral blood, secretion IgM is attenuated in B cells located in TLS of pancreatic ductal adenocarcinoma [228].

#### 4.1.4 T Cell

**FIGURE 4:**
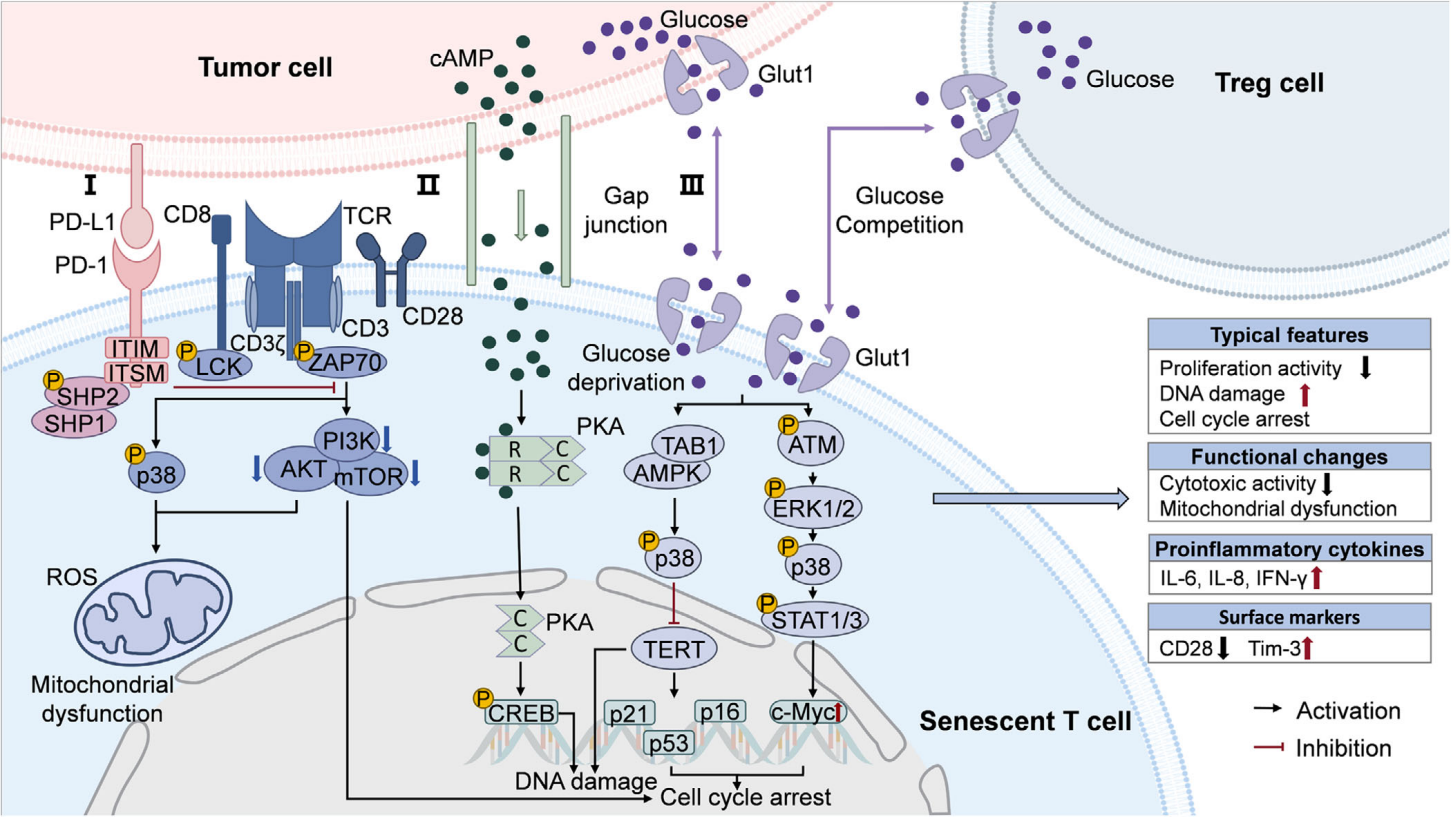
Molecular pathways involved in tumor‐associated T cell senescence. T cell senescence is driven by tumor through multiple interconnected pathways: (I) PD‐1/PD‐L1‐mediated diminished CD28/TCR signaling, leading to p38 signal activation as well as mitochondrial dysfunction. (II) Tumor‐derived cAMP is directly transferred to T cells through gap junctions, triggering cAMP–PKA–CREB axis and DNA damage response (DDR). (III) Glucose shortage also promotes ATM‐related DDR and senescence‐related signaling pathways in T cells.

Originating from primordial T cell precursors, mature T lymphocytes are broadly categorized into CD8^+^ and CD4^+^ T cell subsets. Generally, CD8^+^ CTLs mediate direct tumor cell killing through granzyme/perforin‐dependent mechanisms, while CD4^+^ T helper (Th) and Treg cells maintain immune homeostasis via cytokine‐mediated regulation. There is significant alteration of T cell profiles during age, featured with a declined proportion and proliferative capacity of CD8^+^ T cells, along with diminished cytokine secretion (e.g., GZMB), cell cycle arrest, and shifted phenotypes (e.g., decreased CD28 and increased Tim‐3 expression) (Figure 4). The age‐related decline in CD8^+^ T cells has been observed in aged individuals across many species including murine and canine models [84, 229, 230]. Under stimulation of antigens, CD8^+^ T cells mediated immune responses can be initially activated in lymph nodes through binding to IL‐4 derived from CD4^+^ T cells, followed by effector differentiation [231]. However, aging‐associated attrition of IL‐4‐producing CD4^+^ T cells directly compromises proliferation of CD8^+^ T cells [232]. In addition to altered abundance and proportion, there are molecular reprogramming events and function defects in senescent T cells. For instance, cell cycle inhibitor proteins (e.g., p16^INK4A^, p21, and p53) are uniformly upregulated [233]. Conversely, CD28, a critical costimulatory molecule in antigen recognition and T cell activation, is markedly downregulated in senescent T cells, disrupting their binding with antigen‐presenting cells to weaken T cell‐mediated cytotoxicity, which has been detected in patients with HIV infection or RA [234, 235]. Furthermore, inhibitory immune checkpoint protein such as Tim‐3 is significantly upregulated in senescent T cells, braking the production and secretion of tumoricidal cytokines, such as IL‐2, TNF‐α, GZMB, and IFN‐γ, as well as restricting cytotoxicity of those senescent CD8^+^ T cells [85, 236].

Beyond aging‐related physiological T cell senescence, TME and other pathologic conditions are confirmed to contributed to T cell senescence through distinct mechanisms. Compared with healthy peripheral blood, a marked reduced population of circulating CD8^+^ T cells is observed in blood from patients with multiple malignancies, including colorectal cancer, Hodgkin lymphoma, breast cancer, and NSCLC [237, 238, 239, 240]. In particular, tumor‐infiltrating T cells are typically presented with classic immunosenescent characteristics, incorporating with impaired production of cytokines (e.g., GZMB) and phenotypic changes (e.g., downregulated expression of CD28 and upregulated expression of Tim‐3). In pancreatic ductal adenocarcinoma, CD8^+^ T cells infiltrated in both tumor tissue or metastatic lymph nodes demonstrate compromised capacity of secreting GZMB, constructing an immunosuppressive TME [241]. Downregulated CD28^+^ T cells have been observed in colorectal cancer, effectively crippling antitumor immune surveillance by disrupting CD8^+^ T cell activation [242]. Not surprisingly, CD28^−^CD8^+^ T cells are found to be abnormally expanded in individuals with NSCLC [243].

Continuous stimulation of tumoral antigens directs to T cell exhaustion through facilitating immune checkpoint factors, which also serves as senescent modulator of T cells. Based on the AI‐mediated trajectory calculation via AI, the dominant role of antigen‐specific epigenetic regulatory patterns in CD8^+^ T cells aging has been revealed [244]. The PD‐1/PD‐L1 axis also represents a prototypical example of this dual regulatory mechanism. Following PD‐1/PD‐L1 ligation, immunoreceptor tyrosine‐based inhibitory motif (ITIM) and immunoreceptor tyrosine‐based Switch motif domains of PD‐1 are phosphorylated, converting Src homology 2‐containing protein tyrosine phosphatase 2 (SHP‐2) into an active conformation. Activated SHP‐2 dephosphorylates CD3ζ and costimulatory signal CD28 to restrain activation of phosphoinositide 3‐kinase (PI3K)/protein kinase B (AKT) signaling pathway, which not only participates in functional failure, but also leads to cycle arrest and senescence in T cells [245]. This is in accordance with the clinical evidence that expression of PD‐L1 is significantly negatively correlated with infiltration of CD8^+^ T cells, such as in ovarian cancer and NSCLC [246, 247]. Contained in extracellular vesicles derived from tumor cells, PD‐L1 activates cAMP‐response element binding protein (CREB) and STAT signaling pathways, which induce high expression of lipid metabolism enzymes in T cells, promoting lipid metabolism to induce T cell senescence [248]. Blocking the binding of PD‐1 and PD‐L1 effectively reverses senescence process of T cells [249, 250]. By the methods of gene knockout or inhibitors, blocking interaction between PD‐L1 and T cells reverses aging state of T cells, through inhibiting CREB and STAT signaling pathways [248]. This phenomenon has also been demonstrated in mice model, where PD‐1 blockade improves cytotoxic capacity and reverses senescent CD8^+^ T cells in old mice [251]. In addition, PD‐1 antibody can also reduce expression of p16 and activate CD8^+^ T cells to improve systematic immunosenescence [249]. Treatment with anti‐PD‐1 therapy limited senescent T cells but recovered expression of CD28 in Hodgkin lymphoma patients [238].

Extensive metabolic reprogramming events in TME also takes charge of senescence of infiltrated T cells, primarily through glucose deprivation and release of cAMP. High glycolytic activity of tumor cells deprives environmental glucose, that hastens senescence of immune cells [252]. Additionally, senescent T cells prefer to produce energy through anaerobic glycolysis, magnifying mitochondrial malfunction and generating ROS to expedite senescent process [253]. Tumor‐secreted TGF‐β drives differentiation of CD4^+^ T cells into Treg cells, which exhibit enhanced glucose uptake and glycolysis capacity, further intensifying glucose scarcity [254, 255]. In addition, metabolites of glycolysis of tumor cells promote senescence of T cells in indirect manners. In a tumor–T cell coculture system, an aging phenotype emerges in healthy donor‐derived human T cells followed with stimulation of tumor‐derived cAMP, including decreased expression of CD28, shortened telomere, increased p53, p21, and p16 [87]. Tumor‐derived cAMP diffuses into T cells through intercellular gap junctions and binds to regulatory subunits of inactive protein kinase A (PKA) tetrimers, resulting in the release and activation of catalytic subunits. The subsequent phosphorylation of CREB activates the PKA–CREB signaling axis, ultimately driving T cell senescence [145, 256]. Other cellular signals like NF‐κB, CCAAT/enhancer‐binding protein (C/EBP‐β), and cGAS–STING are also critical in inducing senescent T cells [257].

### 4.2 Content‐Dependent SASPs

Immunosenescence is characterized not only by intrinsic cellular aging of immune cells but also by excessive production of SASP factors that significantly influence microenvironment remodeling and disease progression (Table 4). On the one side, both senescent cells and stromal cells (e.g., macrophages; and cancer‐associated fibroblasts, CAFs) produce and release SASP factors into microenvironment [258]. On the other side, senescent or dysfunctional immune cells exhibit diminished capacity to eliminate these factors, leading to progressive SASP accumulation [259]. These accumulated SASPs demonstrate context‐dependent impact under distinct disease circumstances [260].

**TABLE 4:** Disease‐associated SASP components and their distinct function during pathology.

SASP category	SASP components	Diseases	Function during pathology	References
Interleukin	IL‐1α	Pancreatic cancer	Promoting tumor progression and immune cell infiltration	[261]
		Breast cancer	Inducing tumor survival factors production	[262]
		Lung cancer	Inhibiting CD8^+^ T cell infiltration	[263]
	IL‐1β	Hair cell astrocytoma	Inducing growth arrest and senescence of tumor cell	[264]
		Pancreatic ductal adenocarcinoma	Inducing T cell infiltration; stimulating angiogenesis; inducing tumor‐associated macrophage infiltration	[265, 266, 267]
		Colorectal cancer	Stimulating angiogenesis; inhibiting T cell proliferation	[268]
		Renal cell carcinoma	Promoting tumor metastasis	[269]
		Breast cancer	Promoting tumor metastasis	[270]
		Intervertebral disc degeneration	Stimulating ECM degradation; accelerating cellular senescence	[271, 272]
		RA	Promoting T cell polarization toward Th17 phenotype; amplifying T cell response	[273]
		IVDD	Promoting ECM degradation and increasing MMP‐2/3 expression; aggravating intervertebral disc degeneration via NF‐κB signaling	[274]
		COPD	Promoting chronic airway inflammation	[275]
		Atherosclerosis	Accelerating vascular inflammation and plaque progression via NLRP3–IL‐1β signaling	[276]
	IL‐6	Breast cancer	Enhancing aging/inflammatory environment; enhancing tumorigenic capacity; promoting angiogenesis and tumor invasiveness; promoting hypoxia tolerance; promoting growth of tumor	[277, 278, 279, 280, 281]
		Pancreatic ductal adenocarcinoma	Inducing production of SASPs	[265]
		Alzheimer's disease	Inducing activation and proliferation of astrocytes	[282]
		Parkinson's disease	Inducing oxidative stress in the endoplasmic reticulum of microglia	[283]
		Pulmonary hypertension	Promoting pulmonary hypertension and pulmonary vascular remodeling	[284]
	IL‐7	Esophageal squamous cell carcinoma	Inhibiting expansion of T cell	[285]
		Hepatocellular carcinoma	Increasing proliferation of CART cell	[286]
		Melanoma	Promoting accumulation of CD8^+^ T cell	[287]
	IL‐8	Breast cancer	Promoting tumor invasiveness; inhibiting infiltration of neutrophils; promoting angiogenesis and tumor invasiveness	[281, 288, 289]
		Melanoma	Stimulating tumor cells proliferation	[290]
		Hepatocellular carcinoma	Promoting activation and polarization of M2 macrophage; promoting tumor growth	[291]
		RA	Increasing senescent synovial fibroblast number; enhancing inflammatory environment	[292]
	IL‐15	Lupus nephritis	Amplify cytokine secretion; enhancing migratory capability of macrophages	[293]
	IL‐21	Systemic lupus erythematosus	Inducing accumulation of age‐associated B cell	[294]
	IL‐33	Retinal degeneration	Inhibiting macrophage activation for suppression of retinal inflammation	[295]
Chemokine	CXCL‐1	Malignant mammary epithelial cells	Promoting tumor cell proliferation	[296]
		Pancreatic cancer	Enhancing T cell infiltration	[265]
		Glioblastoma multiforme	Promoting tumor progression; promoting MDSCs recruitment	[297]
		Pancreatic ductal adenocarcinoma	Promoting MDSCs recruitment	[298]
	CXCL‐10	Pancreatic ductal adenocarcinoma	Enhancing T cell infiltration	[265]
		Glioblastoma	Activating T lymphocytes; recruiting T cell	[287, 299]
		Lung cancer	Increasing CD8^+^ T cell infiltration	[300]
		Hepatocellular carcinoma	Recruiting T cells into TME	[301]
		Pulmonary hypertension	Inducing pulmonary artery endothelial cell apoptosis	[302]
		RA	Driving CXCR3⁺ immune cell infiltration in synovial fluid and synovium and enhancing joint destruction	[303]
	CXCL‐11	Breast cancer	Promoting tumor invasiveness	[304]
		Advanced prostate cancer	Promoting epithelial–mesenchymal transformation	[305]
		Colorectal cancer	Enhancing CD8^+^ T cell and DCs infiltration	[306]
	CXCL‐12	Prostate cancer	Promoting tumor progression; promoting angiogenesis	[307, 308]
		Hepatocellular carcinoma	Inhibiting infiltration of CTLs; promoting MDSCs recruitment; inhibiting differentiation of CD8^+^ naive T cell into CD8^+^ cytotoxic T cell	[309, 310]
		Melanoma	Promoting CD8^+^ T cell to migrate out of tumor	[311]
		Glioblastoma	Increasing tumor‐associated myeloid cell population	[312]
		RA	Promoting T cell migration and maintaining inflammatory microenvironment in synovium/endothelium; promoting disease activity	[313, 314]
	CCL2	Pancreatic ductal adenocarcinoma	Enhancing T cell infiltration	[265]
		Hepatoma carcinoma	Recruiting macrophages to clear senescent precancerous cell	[315]
		Cardiac fibrosis	Promoting release of TGF‐β and chronic fibrosis	[316]
		RA	Recruiting macrophages and promoting disease activity	[292, 317]
		Atherosclerosis	Recruiting monocytes into plaques, driving chronic vascular inflammation	[318]
	CCL5	Melanoma cells	Recruiting TILs to eliminate cancer cell	[319]
		Non‐small cell lung cancer	Promoting M2‐like TAM to infiltrate tumor tissue	[320]
		Breast cancer	Promoting CD8^+^ T cell recruitment	[321]
		Pulmonary arterial hypertension	Promoting proliferation and migration of pulmonary arterial smooth muscle cell	[302]
	CCL11	Multiple sclerosis	Inhibiting oligodendrocytes maturation; inhibiting myelin regeneration	[322]
	CCR2	Colorectal cancer	Promoting tumor cell proliferation and invasion; inhibiting CD8^+^ T cell activation	[323, 324]
		RA	Served as a receptor for CCL2 mediating monocyte recruitment and inflammation amplification	[325]
Growth factor	CTGF/IGFBP‐rP2	Prostate cancer	Promoting angiogenesis and tumor progress	[326]
	MIC‐1/GDF15	Colorectal cancer	Promoting proliferation and metastasis of tumor cell	[327]
	TGF‐β	Hepatocellular carcinoma	Inducing cell cycle arrest and senescence; promoting tumor cell invasiveness	[328]
		Brain glioma	Weakening killing effect of NK cell	[329]
		Cardiac fibrosis	Activating SMAD3 signaling pathway; promoting secretion and deposition of ECM; inducing fibrosis	[330]
	HGF	Pancreatic cancer	Promoting tumor cell invasiveness	[331]
		Colorectal cancer	Inhibiting tumor‐killing neutrophils invasion	[332]
		Non‐small cell lung cancer	Promoting tumor proliferation and invasion; promoting angiogenesis	[333]
Others	MMP3	Bladder cancer	Promoting tumor cell invasiveness and metastasis	[334]
		Pancreatic ductal adenocarcinoma	Promoting T cell infiltration	[265]
		IVDD	Promoting ECM degradation and degeneration of intervertebral disc	[335]
	MMP2	Prostate cancer	Promoting tumor progress	[336]
		Pancreatic ductal adenocarcinoma	Promoting T cell infiltration	[265]
		Multiple sclerosis	Damaging myelin sheath and axons	[337]
	MMP1	Breast cancer	Enhancing mitosis of epithelial cells; increasing tumorigenicity	[338]
		Large cell carcinoma	Promoting tumor cell invasiveness	[339]
	MMP9	Renal fibrosis	Increasing neutrophils and other inflammatory cell infiltration; promoting kidney fibrosis	[340]
		Hypoxic pulmonary hypertension	Producing a proinflammatory microenvironment	[341]
	TNF‐α	Pancreatic cancer	Blocking redistribution of CD8^+^ T cell into tumor	[342]
		Lung cancer	Promoting immune surveillance function and tumor killing effect of NK cell	[343]
		Colorectal cancer	Inhibiting tumor growth and metastasis	[344]
		Breast cancer	Decreasing tumor aggressiveness	[345]
		Intervertebral disc degeneration	Inducing intervertebral disc cell apoptosis	[346]
		Parkinson's disease	Enhancing endothelial release of CXCL10; disrupting blood–brain barrier	[347]
		RA	Increasing other SASPs (e.g., IL‐6, IL‐8, CXCL8, and MMP3) numbers; promoting chronic inflammation in RA	[292]
	IFN‐γ	Melanoma	Enhancing resistance of tumor cell to NK cell; promoting PD‐L1 expression on tumor cell; stimulating N1 type neutrophils to kill tumor cell by releasing NO; killing tumor cell directly	[348, 349, 350, 351]
		Squamous cancer	Promoting T cell infiltration	[352]
		Breast cancer	Inducing tumor stem cell transformation	[353]
	GROα	Parkinson's disease	Impairing synaptic transmission and cognitive function	[354]
		Breast cancer	Stimulating precancerous cell growth	[355, 356, 357]
		Melanoma	Stimulating precancerous cell growth	[358]
	Galectin‐9	Melanoma	Promoting T cell and monocyte apoptosis	[359, 360]
	E‐cadherin	Melanoma	Promoting tumor cell metastasis	[361]
	COTL1, ENO1, PRDX2	Hepatocellular carcinoma	Promoting tumor cell proliferation	[362]
	Extracellular vesicles (EVs)‐associated EphA2	Breast cancer; ovarian cancer	Promoting tumor cell proliferation via reversing signaling of EphA2 binding to ephrin‐A1, accompanied by a proinflammatory SASP milieu (e.g., IL‐6, IL‐8)	[363]

This table displays disease‐associated SASP components as categorized by cytokine subtypes, and their distinct function during pathology.

*Abbreviations*: CART cell, chimeric antigen receptor T cell; CCL, C‐C motif chemokine ligand; CCR, C‐C motif chemokine receptor; COPD, chronic obstructive pulmonary disease; COTL1, coactosin‐like protein 1; CTGF, connective tissue growth factor; CTLs, cytotoxic T lymphocytes; CXCL, C‐X‐C motif chemokine ligand; CXCR3, C‐X‐C motif chemokine receptor 3; DCs, dendritic cells; ECM, extracellular matrix; ENO1, alpha‐enolase/enolase 1; EV, extracellular vesicle; GDF15, growth differentiation factor 15; GROα, growth regulated oncogene α; HGF, hepatocyte growth factor; IFN‐γ, interferon‐gamma; IGFBP, insulin like growth factor binding protein; IL, interleukin; IVDD, intervertebral disc degeneration; MDSC, myeloid‐derived suppressor cell; MIC‐1, macrophage inhibitory cytokine‐1; MMP, matrix metalloproteinases; NK, nature killer cells; PD‐L1, programmed cell death ligand 1; PRDX2, peroxiredoxin 2; RA, rheumatoid arthritis; SASP, senescence‐associated secretory phenotype; TAM, tumor‐associated macrophage; TGF‐β, transforming growth factor beta; TIL, tumor infiltrating lymphocyte; TME, tumor microenvironment; TNF‐α, tumor necrosis factor‐α.

Accelerating evidence unearths the proinflammatory and immune‐suppressive effects of SASPs in the interplay between cancer and TME through cell‐type specific mechanisms. The infiltration and activation of various immune cells such as NK and T cells can be inhibited by numerous SASPs, impeding maturation of immune cells and release of cytotoxic molecules. For example, senescent CAFs in breast cancer directly inhibit maturation and infiltration of NK cells by extracellular matrix components (e.g., collagens, MMPs) [364]. MMPs or TGF‐β inhibits expression of activator receptor NKG2D on NK cells, causing damaged immunosurveillance and cytotoxicity of NK cells [365, 366]. By binding to CD47 on NK cells, melanoma‐derived thrombospondin‐1 suppresses generation of GZMB and IFN‐γ to cripple cytotoxic effect of NK cells [367]. Similarly, production of GZMB and IFN‐γ can be blocked by TGF‐β–Smad signals in CD8^+^ T cells [368]. Major proinflammatory SASPs also exert antitumor effect by inducing secondary cell death in tumor cells directly, while some SASP factors act to augment infiltration of immune cells and immune reaction. For instance, by attaching to C‐C motif chemokine receptor (CCR)2 receptor on NK cells, CCL2 promotes recruitment of NK cells. In murine lung cancer models, tumor cell‐secreted TNF‐α activates NF‐κB signaling, potentiating their tumoricidal activity in lung cancer models [343, 369, 370]. CSF‐1, CCL2, and IL‐15 are found to enhance the clearance of macrophages on hepatocellular carcinoma (HCC) cells [371]. In addition, chemotactic SASPs including GM‐CSF, macrophage colony‐stimulating factor (M‐CSF), and TGF‐β drive a large influx of MDSCs and Treg cells to build immunosuppressive environment [372, 373, 374]. MDSCs take up extracellular vehicles released by tumor cells to activate cGAS–STING–NF‐κB signaling pathway and release IL‐6 [375]. Besides with expressing heterogeneous immunological checkpoint molecules to brake immune reaction of T cells or NK cells (e.g., PD‐L1, Tim‐3), intratumoral MDSCs also secrete SASPs like indoleamine 2,3‐dioxygenase and Arg1 to recruit Treg cells [376, 377, 378, 379].

Distinct SASP components have also been identified to drive pathogenesis across diverse non‐neoplastic diseases through cytokine‐specific mechanisms. Abnormal overproduction of IL‐6, IL‐8, and TNF‐α are overlapped in almost diseases, prominently in degenerative (e.g., AD, osteoarthritis) and cardiovascular pathologies (e.g., atherosclerosis) [282, 380, 381]. Numerous cytokines or chemokines (IL‐1β, IFN‐γ) have been confirmed to synergistically amplify inflammatory signals in other pathogenic conditions like autoimmune disorders. A part of SASP factors with protease properties (e.g., MMPs) participated in destroying extracellular components to promote tissue damage directly [382].

## 5 Role of Immunosenescence in Disease Vulnerability and Pathogenesis

In recent decades, the incidence of age‐related disorders markedly rises, particularly cardiovascular and neurodegenerative diseases. Beyond specific organ pathology, systemic diseases such as autoimmune diseases, infectious diseases, and cancer are becoming more prevalent. On the one side, due to biological clock, almost organs enter an irreversible senescent state with the increase of organ‐specific biological age. On the other side, chronic inflammation formed by immunosenescence disrupts the integrity of stem niche to block regenerative ability of diverse tissues and aggravates their susceptibility to aging‐related tissue damage. Under the systematic multiorgan aging, metabolic coupling of organs is broken, particularly the imbalance of “brain–immune system axis,” exhibiting a unique aging pattern that is highly coordinated with immunosenescence during various pathogenic conditions.

### 5.1 Cancers

#### 5.1.1 Immunosenescence in Tumorigenesis and Metastasis

Tumor cells have evolved a variety of mechanisms to actively evade immune surveillance, such as downregulation of immunogenicity, secretion of immunosuppressive factors, and metabolic competition. Correspondingly, the restricted immune response in the process of immune senescence also passively promotes tumor immune escape. Immunosenescence fuels formation of malignant microenvironment, intensifying tumorigenesis in a self‐perpetuating cycle. According to epidemiological data, there is a higher risk of cancer in middle‐aged and elderly populations than adolescents or newborns [383, 384]. In orthotopic lung adenocarcinoma and colorectal carcinoma models, there is a larger tumor burden in aged mice than younger controls, revealing a stronger supporting effect on tumor growth in aged organisms [385]. It is generally believed that not only aging‐accumulated oncogenic mutations raise the risk of cancer, but also the dynamic evolved multidimensional immunosenescence plays a vital role in regulating tumor development.

Age‐related decline in immune cell quantity and function, particularly in T cell populations, significantly compromises immunosurveillance and cytotoxic capacity, thereby facilitating tumor development [386]. Comprehensive analyses of tumor‐associated characteristics reveal an inverse correlation between aging and T cell abundance, concomitant with increased cancer incidence [216]. Additionally, senescent immune system promotes chronic inflammation and angiogenesis by producing SASPs such as IL‐1, IL‐6, TNF‐α, and IFN‐γ contributing to creating a tumor‐tolerant microenvironment [119]. For example, senescent T cells and HSCs increase the secretion of IL‐1α of IL‐1β and recruitment of MDSCs and neutrophils, suppressing infiltration and function of CD8^+^ T and NK cells [263, 387, 388]. Furthermore, once bound with IL‐1R, IL‐1α triggers nuclear translocation of NF‐κB, and sequentially activates transcription of inflammatory mediators including IL‐6 and IL‐8 [389]. Senescent cell‐derived IL‐8 activates macrophages, further promoting production of inflammatory cytokines and infiltration of macrophages to be involved in the occurrence of HCC [291]. Under synergistic stimulation of extracellular IL‐6 and IL‐8, breast cancer cells amplify secretion of IL‐6 and IL‐8 to intensify tumor‐related inflammatory environment by self‐enhancing feedback [277].

SASP components (e.g., IL‐1 and IL‐8) also advertise tumor neovascularization, facilitating oxygen and nutrients supply to meet the requirement of rapid tumor proliferation [390]. For example, IL‐1β enhances angiogenesis by upregulating proangiogenic factors while concurrently suppressing angiogenesis inhibitors in senescent fibroblasts [391]. Similarly, a larger extent of angiogenesis is observed in mice injected with breast cancer cells with elevated IL‐8 expression [289]. Conversely, targeted ablation of senescent macrophages and associated SASPs in lung cancer models lowers tumor vascular density and overall tumor burden, effectively attenuating disease progression [57].

Accompanied with in situ growth of tumors, other malignant phenotypes such as invasion and metastasis occur in most tumors. Acquire invasive capacity through epithelial–mesenchymal transition (EMT) is regarded as the initial metastatic event, before migrating into adjacent blood or lymphatic vessels to enter circulation as circulating tumor cells (CTCs) [392]. Changes of cellular senescence pathways in tumor cells are involved in the regulating tumor metastasis. As revealed by tumor samples from relapsed cervical carcinoma patients, there is opposing altered expression of senescent markers and SASP factors between patients with local and distant relapses following radiotherapy [393]. In contrast, abundant SASP factors especially members of IL family are effective in stimulating EMT. For instance, IL‐6 and IL‐8 secreted by breast cancer cells induce EMT and promote acquisition of more aggressive phenotypes [277].

Once entering circumstance, substantial apoptosis occurs in CTCs due to mechanical stress, redox pressure, immune surveillance and tumor killing effect [392, 394]. Aged immune system, particularly marked with significantly declined peripheral CD8^+^ and CD4^+^ T cells, is commonly fatigued to recognize or clear CTCs [395]. During melanoma metastasis, a Wnt antagonist, secreted frizzled‐related protein 2 (sFRP2), can be secreted by senescent fibroblasts to enhance resistance to redox stress of tumor cells [396]. Subsequently, a unique premetastatic niche (PMN) conducive to colonization in target organ is generated, which is under regulation of both tumor cells at primary site and CTCs through paracrine effect [397]. Senescence‐related immune system alterations are reported to shift the microenvironment toward a PMN. For example, there is upregulated expansion of γδT cells and protumor neutrophils in aged mice with melanoma, which limits cytotoxicity of CD8^+^ T cells to build a metastasis‐permissive microenvironment [398].

After reaching target organs, disseminated tumor cells (DTCs) enter a state of temporary dormancy, maintained by immune surveillance and limited blood supply, while retaining the potential for reactivation upon immune system compromise [399]. Immunosenescence declines surveillance ability of CD8^+^ T and NK cells, disrupting the delicate equilibrium between dormant DTCs and the immune microenvironment. This imbalance is evidenced in aged murine models, where bone marrow exhibits reduced frequencies of dormant DTCs concomitant with enhanced tumor cell proliferation [400]. As for hepatic metastatic model, activated HSCs (aHSCs) suppress proliferation of NK cells by secreting CXCL12 to terminate dormancy of DTCs [401]. Moreover, a series of anti‐inflammatory factors (e.g., TGF‐β) are synthesized to recruit immunosuppressive cell populations (e.g., Treg cells, MDSCs), which exacerbates immunosenescence and reinforces an immunosuppressive niche [402]. This reciprocal tumor–immune interactions accelerates tumor metastatic colonization and development.

#### 5.1.2 Immunosenescence in Therapeutic Response or Resistance of Cancer

Recent advances in innovative tumor therapies, particularly immunotherapy, have revolutionized conventional cancer treatment paradigms encompassing surgery, chemotherapy, and radiotherapy. Nevertheless, clinical outcomes remain suboptimal for most patients. Multidimensional computational profiling enables quantitative prediction of therapeutic response and resistance development, thereby optimizing treatment strategies. Patients with elevated immune senescence indices (ISI) scores exhibit poor prognosis across malignancies, characterized by suppressed antigen presentation and ligand‐receptor downregulation [27]. Furthermore, peripheral blood biomarkers of immunosenescence complement traditional tumor assessments (e.g., histopathology and imaging), offering minimally invasive dynamic monitoring. For instance, regression analysis of lymphocyte subsets in 332 nasopharyngeal carcinoma patients identified CD28 downregulation in senescent CD8⁺ T cells as a stratification biomarker and independent prognostic factor for progression‐free survival and distant metastasis‐free survival, longitudinally validated to predict suboptimal radiotherapy response [50].

Critically, immunosenescence contributes to chemoradiotherapy failure and increased posttreatment recurrence risk. High‐dose therapy damages HSCs, depleting immune cell reserves and compromising systemic immunity. Treatment pressure upregulates immunosenescence across malignancies. For example, peripheral T cells in breast cancer exhibit transient downregulation of CD28/CD27/leucine rich repeat neuronal protein 3 (LRRN3) expression postchemotherapy, while TGF‐β induced by chemoradiotherapy promotes senescent CD27^−^CD8⁺ T cells in cervical cancer [53]. These cells directly associate with adverse outcomes and demonstrate elevated frequencies in postchemoradiotherapy recurrent cases [61].

Almost immunotherapeutic strategies targeting immunosuppression to restore antitumor immunity, particularly immune checkpoint blockade (ICB) via small molecules, are confirmed to exhibit age‐dependent efficacy disparities. AI‐based modeling reveals that elevated senescence scores correlate significantly with immune‐suppressive cells (e.g., Treg cells and MDSCs) infiltrated within tumors, whereas linked with diminished responses after ICBs treatment [25]. In elderly patients with advanced NSCLC, impaired ICB efficacy is attributed to T cell senescence in the TME. Terminally differentiated T cells accumulated in these patients demonstrate coupregulation of PD‐1/CTLA‐4/T cell Ig and ITIM domains, which disrupts immune synapse formation [403]. Similarly, nonresponding melanoma patients exhibit higher frequencies of senescent CD4⁺ and CD8⁺ T cells (marked by reduced CD27/CD28 expression) compared with ICI responders, an observation validated in murine colorectal cancer models where PD‐L1 antibody fails to suppress tumor growth in aged mice [404]. Depleting senescent cells with ABT263 (navitoclax) enhances intratumoral CD8⁺ T cell infiltration and prolongs survival [309]. Therefore, resistance to ICBs can be attributed to both the off‐target effects generated from heterogeneity of senescent cell subpopulations and compensatory upregulation of PD‐L1 through immunosenescence‐driven pathways [405, 406]. For instance, senescent T cells overproduce IFN‐γ, promoting internalization of PD‐L1‐rich exosomes that evade lysosomal degradation via chaperone‐mediated autophagy, thereby inducing adaptive resistance [248]. Furthermore, monocyte‐triggered IFN‐γ upregulates the PD‐L1 axis, enabling endogenous monocyte‐derived PD‐L1 to evade conventional antibody targeting and perpetuate treatment resistance [407].

### 5.2 Cardiovascular Diseases

Among numerous age‐related diseases, cardiovascular diseases (CVDs) account for the leading cause of human mortality. CVDs, primarily comprising atherosclerosis and heart failure, involve complex pathophysiological interactions among metabolic, immune, and neural regulatory disturbances within vascular and cardiac tissues, ultimately leading to cardiovascular dysfunction and pathological cardiac structural remodeling. Among multifactorial influences on CVD, multidimensional biological senescence is recognized as a principal impetus, exhibiting distinctive association patterns with CVDs. As confirmed in large‐scale clinical cohort studies, risk of CVDs in individuals aged 85–89 years are 441–630‐fold higher compared with those aged 25–29 years [408]. In pathological states such as hypertension, atherosclerosis, and arrhythmias, majority of senescent adaptive immune cells (e.g., CD8^+^ T cells and B cells) typically promote disease progression by driving inflammatory processes and facilitating accumulation of characteristic pathological changes, including foam cell formation and destabilization of plaque [409]. For example, senescent CD8^+^ T cells, labeled with CD57^+^KLRG1^+^ CD27^−^CD28^−^, are enriched in both peripheral circulation and atrial myocardium of patients with atrial fibrillation (AF), promoting AF onset and postablation recurrence by disrupting intracellular calcium homeostasis [410].

Generally, atherosclerosis is regarded as the pathological basis of many CVDs, manifested by initial formation of deposited lipid plaques in the intima of arteries, and subsequent thrombosis and blood supply disorders. In the early stage of atherosclerosis, senescent T cells contribute to a proinflammatory environment by continuously secreting inflammatory cytokines (e.g., IL‐6 and TNF‐α), exacerbating endothelial injury and pathological vascular remodeling, thereby augmenting risk of atherosclerosis as well as major adverse cardiovascular events [411, 412]. Upon establishment of atherosclerosis, these proinflammatory factors further escalate. They not only sustain the upregulation of vascular endothelial adhesion molecules (e.g., vascular cell adhesion molecule 1 [VCAM‐1] and intercellular cell adhesion molecule‐1 [ICAM‐1]), facilitating immune cell infiltration that directly impairs endothelial function, but also exacerbate the chronic low‐grade inflammatory state [413]. For example, CCL2, IL‐1, MMP12, and MMP13, derived from senescent macrophages in atherosclerosis, maintain monocytes‐mediated vascular inflammation and promote lipid accumulation and plaque instability, thereby inducing myocardial infarction [414].

The systematic chronic inflammation also profoundly impacts on lipid and glucose metabolism, intensifying energy crisis during CVDs. Serving as the dominant immune cell type in protecting vessels from atherosclerotic plaques, macrophages regulate cholesterol homeostasis by phagocytosing oxidized low‐density lipoproteins, thereby preventing excessive intracellular lipid accumulation and subsequent foam cell formation. However, protection of macrophages can be damaged by aging‐induced metabolic disorder, with excessive intracellular lipid accumulated and subsequently transformed into foam cells. Macrophages also induce mitochondrial dysfunction and increase ROS generation in cardiomyocytes by polarizing toward M1, further exacerbating myocardial injury. Depleting those aged macrophages in myocardial infarction by administration of alpha‐lipoic acid, alleviate myocardial cell damage and improve clinical prognosis as expected [415].

### 5.3 Neurodegenerative Diseases

Neurodegenerative diseases are typically characterized by progressive loss of neuronal structure and function, leading by AD, PD, and amyotrophic lateral sclerosis. The prevalence and incidence of these diseases rise significantly among the elderly, constitute the main cause of dementia, disability and even death in this population. According to the *2021 World Alzheimer's Report*, over 55.2 million people globally are affected by dementia [416]. However, the tardy progress in research has left a gap in effective interventions to reverse the course of these diseases. Contrary to the traditional focus on neuronal pathology, emerging evidence indicates that immunosenescence is not merely a bystander but a critical driver of neuroinflammation and related disease progression. Dysfunctional senescent immune cells, particularly central nervous system (CNS)‐resident microglia, create a chronic inflammatory environment, interacting with misfolded proteins to form a vicious cycle. This interactive pattern has profoundly renewed our understanding of neurodegenerative mechanisms and redirected potential intervention strategies.

#### 5.3.1 Alzheimer's Disease

As the most prevalent age‐related neurodegenerative disease, the incidence of AD increases dramatically along with age. There are approximately 50 million individuals are affected by AD globally, with the highest number of patients reaching 9.83 million in China [417]. The major clinical syndromes of AD are manifested as cognitive and behavioral impairments. Current mainstream theory of AD centers on the abnormal accumulation of amyloid‐beta (Aβ) and tau protein pathology, which is characterized by accumulating extracellular Aβ plaques and intracellular neurofibrillary tangles mainly composed of hyperphosphorylated tau protein, accompanied by excessive activation and proliferation of astrocytes. In addition to the well‐documented genetic and environmental factors, disruption of CNS homeostasis and immunosenescence‐related chronic neuroinflammation have also been confirmed to act parallelly in AD.

Microglia are the resident macrophages of CNS, widely involved in CNS immune surveillance, inflammatory responses, and neural development. In AD, microglia serve as the pivotal cells responsible for eliminating Aβ, whereas significant impaired phenotypes of microglia appear during AD. For example, the downregulation of Aβ phagocytic receptors (e.g., scavenger receptor class A member 1, SCARA1; CD36) and the upregulation of inhibitory receptors (e.g., CD33) contribute to impaired phagocytic function of microglia [418]. Occurrence of lysosomal dysfunction in microglia fails to degrade Aβ [419]. Compared with healthy mice, microglia of AD mice display typical aging phenotypes (increased expression of p16^INK4A^, p21, and p53) [420]. These senescent characteristics of microglia such as cell cycle arrest and the expression of p16^INK4A^ and SA‐β‐gal seem to be pathological response after uptake of hyperphosphorylated tau protein (hp‐tau) [421]. Accumulated intracellular Aβ has also been reported to activates p53, resulting in DNA damage and aging of microglial aging [422].

Aging immune cells, especially microglia and T cell, are fatigue to promptly remove neurotoxic substances. Instead, they are modified into proinflammatory phenotype to secret a cluster of SASP cytokines such as IL‐1β, IL‐6, and TNF‐α to exacerbate neuronal damage and synaptic dysfunction. Senescent microglia facilitate the release of proinflammatory factors (e.g., IL‐6 and IL‐8) through lactate–H3K18la–NF‐κB axis, promoting the aggregation of Aβ protein and contributing to neuronal dysfunction and death [423]. IL‐1α and TNF can expand the neuronal damage by transforming adjacent astrocytes into proinflammatory, neurotoxic A1‐type cells, and accelerating the progression of AD [424, 425].

#### 5.3.2 Parkinson's Disease

PD is the second most common neurodegenerative disease after AD, affecting approximately 8.5 million people globally and especially in individuals 60 years old [426]. The main clinical symptoms of PD are motor symptoms (e.g., muscle stiffness, resting tremor, bradykinesia, and postural instability) and nonmotor symptoms (e.g., sleep disorders, hallucinations, and dementia) [427]. Pathological hallmark of PD consists of progressive degeneration of dopaminergic neurons in the substantia nigra pars compacta. Meanwhile, misfolded α‐syn is aggregated to form insoluble Lewy bodies, reducing astrocytic uptake of neurotoxic substance to induce neuronal death [428]. In addition to genetic and environmental risk factors, the chronic inflammatory environment mediated by immunosenescence have also been confirmed to play a critical role in PD.

Excessive neurotoxic substances like α‐syn are supposed to be removed by microglia to maintain CNS homeostasis [429]. However, aging‐related morphological changes of microglia in the substantia nigra of PD patients such as reduced branching impairs their ability of clearing neurotoxic substances (e.g., α‐syn) [430]. Accumulated α‐syn interacts with toll‐like receptor (TLR)2 and TLR5 receptors in microglial, triggering activation of NOD‐like receptor family pyrin domain containing 3 (NLRP3) inflammasome, preventing α‐syn uptake and degradation as a feedback [431]. Similar to the situation of AD, senescent microglia also transform into proinflammatory phenotypes, releasing cytokines such as TNF‐α and IL‐1β creating a chronic inflammatory microenvironment to accelerate PD progression. For instance, in aging mice, reduced expression of the triggering receptor expressed on myeloid cells‐2 gene in microglia induces a shift from an anti‐inflammatory to a proinflammatory phenotype, enhancing neuroinflammation in the substantia nigra–striatum pathway [432]. Proinflammatory microglia release TNF‐α, IL‐1β, and IL‐6 via TLR4/NF‐κB pathway, directly damaging dopaminergic neurons in the substantia nigra. Mitochondrial complex I dysfunction in neurons can be induced by proinflammatory factors as well, increasing production of ROS and oxidative stress‐induced neuronal death [433].

Senescent T cell appears to promote PD via compromising the blood–brain barrier (BBB). BBB stands for restricting the entry of peripheral immune cells into brain, protecting dopaminergic neurons from immune‐mediated damage. However, adhesion molecules (lymphocyte function‐associated antigen 1 and very late appearing antigen‐4), overexpressed in senescent T cells bind to vascular endothelial ligands (ICAM‐1 and VCAM‐1), disrupting the integrity of BBB to permit transmigration of peripheral T cells into the substantia nigra [434]. Once infiltrated within brain, perforin/ GZMB derived from those CD8⁺ T cells directly induce apoptosis of dopaminergic neurons and accelerating disease progression [435].

### 5.4 Autoimmune Diseases

Autoimmune diseases encompass a broad pathological spectrum characterized by loss of self‐antigen tolerance, wherein dysregulated immune responses targeting self‐antigens drive tissue injury. These diseases includes both organ‐specific disorders and systemic conditions, such as multiple sclerosis (MS), SLE, and RA. Epidemiological studies report a global autoimmune disease incidence of 0.09% and prevalence ranging from 7.6 to 9.4% [436]. Conventionally viewed as manifestations of immune hyperactivation, these diseases paradoxically exhibit increased incidence in the elderly population—including RA, primary Sjögren's syndrome, and autoimmune bullous diseases—despite the generalized decline in immune competence known as immunosenescence. This apparent contradiction indicates that immunosenescence extends beyond simple functional attenuation in autoimmune pathogenesis. Rather, it orchestrates multifaceted transformation mechanisms that subvert the immune system from a defensive role into a potential “self‐aggressor” through complex mechanisms, thereby contributing to the pathological evolution of autoimmunity.

MS is defined as an immune‐mediated inflammatory demyelinating disorder of the CNS, with an incidence of 0.235 per 100,000 person‐years in China [437]. The major clinical manifestations of MS arise from multifocal cerebral and spinal cord lesions, leading to paresthesia, appendicular weakness, and motor deficits. Although MS etiology remains incompletely defined, substantial evidence implicates immune dysregulation as central to MS pathogenesis [438, 439]. Aberrant activation of autoreactive lymphocytes (e.g., T cells) triggers subsequent inflammatory cascades that disrupts BBB integrity. These activated T cells subsequently migrate into CNS area, releasing proinflammatory cytokine that drive demyelination [440].

Immunosenescence exacerbates disease progression of MS through a dual mechanism of myelin destruction and impaired regeneration. T cells in CNS of MS patients exhibit aged‐related characteristics, such as an inverted ratio of CD4^+^/CD8^+^ T cells and telomeric attrition. Senescent T cells produce proinflammatory factors (e.g., IFN‐γ, IL‐17) that perpetuate neuroinflammation, damaging the BBB and attacking myelin [441, 442, 443, 444]. Moreover, age‐associated mitochondrial dysfunction severely impairs the differentiation ability of oligodendrocyte precursor cells, hindering remyelination to neural repair [445]. Dysregulation of innate and adaptive immunity accelerates conversion of demyelinating lesions to irreversible neurodegeneration. It also compounds therapeutic complexity via compromised anti‐infective immune responses. Through qualitative functional shifts in immune cells, immunosenescence establishes a self‐reinforcing “senescence–inflammation–immune dysregulation” axis, namely, switching the defensive states into autoaggressive states. This pathogenic hub underlies the accelerated progression and treatment refractoriness of autoimmune diseases (e.g., RA, MS) in aging.

Analogous immunosenescent phenomenon manifests in and affect RA, a prevalent systemic autoimmune disorder affecting 0.5–2% of the global population. RA typically peaks in incidence between the fourth and sixth decades of life and exhibits a 3:1 female‐to‐male predominance [446]. Hallmark musculoskeletal features of RA include joint inflammation (synovitis), tendinous sheath inflammation (tenosynovitis), and extracapsular manifestations, such as rheumatoid nodules [447]. Compelling evidence now positions immunosenescence as a key pathogenic driver of RA. For example, T cells in advanced RA display aging‐associated markers, including downregulated CD28 expression and telomere attrition [448]. These senescent immune cells secrete copious SASP factors (e.g., TNF‐α, IL‐6, MMPs), establishing a chronic inflammatory milieu that triggers and perpetuates synovial inflammation, ultimately driving bone erosion. Within this environment, immunosuppressive Treg cells become functionally impaired while proinflammatory Th17 cells undergo enhanced differentiation. Imbalanced Treg cells/Th17 amplifies inflammation and tissue destruction, ultimately propagating RA synovial lesions [448, 449].

### 5.5 Infectious Diseases

According to the *2021 Global Burden of Disease Study*, infectious diseases rank among the top three causes of age‐standardized mortality [450]. Age‐associated declines in immune adaptability, particularly dysfunctional antibody production and thymic involution leading to naive T‐cell depletion, heighten susceptibility to emerging pathogens, such as HIV [451]. Moreover, accumulated senescent immune cells also disrupt immune homeostasis toward persistent pathogens, facilitating reactivation of latent infections including tuberculosis (TB) and varicella‐zoster virus (VZV) [452, 453]. Reciprocally, chronic pathogen exposure generates persistent antigenic stress, fostering a proinflammatory milieu that accelerates immunosenescence, establishing a vicious cycle of infection and immune dysfunction.

HIV‐induced acquired immunodeficiency syndrome (AIDS) remains a major global chronic infectious disease, with approximately 39.9 million people worldwide live with HIV infection [454]. HIV virions target follicular DCs in lymph nodes and germinal‐center CD4⁺ T cells, driving progressive immune depletion through direct and indirect cytotoxic effects on CD4⁺ T lymphocytes and macrophages. HIV infection can also generate immunosenescence, as evidenced by epigenetic aging markers. Recent cohort data reveal that HIV infection increases DunedinPACE acceleration, corresponding to a +7.2‐year elevation in biological age [455]. Peripheral blood mononuclear cells (PBMCs) from AIDS patients exhibit canonical senescence signatures, including a reduced proportion of naïve CD8⁺ T cells and shortened telomeres in B cells, CD4⁺ T cells, and CD8⁺ T cells [456]. Appearance of these characteristics is mainly attributed to impaired function of HSCs and differentiation of HSCs toward lymphoid lineage caused by HIV infection. By hijacking stromal cell‐derived factor 1–CXCR4 axis essential for HSC homing, HIV directly infects HSCs and impairs their colony‐forming capacity in vitro, subsequently disrupting the myeloid/lymphoid differentiation [457, 458]. Meanwhile, progressive loss of CD4^+^ T cells inverts the ratio of CD4⁺/CD8⁺ T cells to blunt immune reconstitution [459]. Selective elimination of senescent CD4⁺ T cells with fisetin, successfully lowers HIV‐DNA loads in lymphoid tissue [460].

During HIV infection, both infected or bystander senescent immune cells secrete SASPs, establishing a chronic inflammatory environment that indirectly fuels viral replication and increases the risk of coinfection. In nonhuman primate models, aged macaques exhibit higher viral loads in secondary lymphoid follicles than young counterparts—a phenotype driven by IL‐6‐enriched inflammaging microenvironments. IL‐6 released by senescent macrophages is also reported to trans‐activate HIV‐long terminal repeat promoter, potentially reactivating latent virus [461, 462]. Furthermore, IL‐6 drives aberrant STAT3 signaling in follicular CD8⁺ T cells, triggering excessive production of GZMB that disrupts follicular architecture and fails to eliminate infected cells, thereby fostering viral replication within germinal centers [463].

TB, the second leading infectious cause of death globally, predominately manifested as pulmonary TB, spreads via airborne *Mycobacterium tuberculosis* (Mtb). In almost infected hosts, Mtb typically establishes latency in immunocompetent hosts, reactivating upon immune decline. Epidemiological data indicate that latent TB infection prevalence escalates with age, affecting 31.38–44.85% of individuals ≥60 years [453]. Age‐related immune surveillance failure facilitates the risk of Mtb reactivation, as well as the occurrence of extrapulmonary TB lesions in the elderly population.

During the early stages of Mtb infection, macrophages serve dual roles in presenting Mtb antigens and becoming primary intracellular niches that sustain bacterial survival postphagocytosis. As a response, macrophages secrete IL‐1, IL‐6, and TNF‐α after phagocytizing Mtb, recruiting immune cells to form granulomas that constrain dissemination. However, the expression of TLR accounting for identifying Mtb is markedly downregulated in senescent macrophages, significantly subverting the initiation of antibacterial immunity [464]. Furthermore, reduced PTEN‐induced putative kinase 1/Parkin pathway in senescent AMs blocks mitophagy and lysosomal Mtb clearance [465, 466]. There are accumulated intracellular cholesterol esters within lipid droplets in senescent macrophages, creating a permissive niche for Mtb persistence [467].

Impaired T‐cell surveillance is a critical driver of Mtb reactivation and aggravative infection. Those infected macrophages are anchored and eliminated by Mtb‐specific CD8⁺ T cells through Fas ligand/Fas‐mediated apoptosis and releasing perforin/GZMB, which is orchestrated by CD4⁺ T‐cell subsets via specialized effector networks. However, CD4⁺ T cells from Mtb‐infected hosts exhibit senescence signatures, including upregulation of p53, reduced production of IL‐2, TNF‐α, and IFN‐γ, resulting in impaired activation of cytotoxic CD8⁺ T cells [468]. T‐cell activation can also be blunted by immunosuppressive SASPs (e.g., TGF‐β and prostaglandin E2, PGE2) secreted by senescent stromal cells, in the manner of blocking DC‐mediated antigen presentation [469, 470]. Mtb‐infected macrophages exacerbate T cell dysfunction by reducing CXCL9/CXCL10 production, curtailing Teff cell recruitment and enabling immune evasion [471]. Consequently, disrupting the inflammaging cascade or rejuvenating senescent T cells represents a therapeutic strategy to restore antimycobacterial immunity and prevent reactivation.

Herpes zoster (HZ) is a quintessential reactivation disease driven by immune decline. Following primary varicella infection, VZV establishes lifelong latency in cranial and dorsal root ganglia in >90% of adults, developing into HZ when latent VZV escapes ganglionic surveillance. Immunosenescence progressively erodes VZV‐specific cell‐mediated immunity to acquiesce in viral reactivation [472]. The incidence of HZ increases sharply as people age, rising 8.3‐fold among individuals ≥60 years [452]. In elderly HZ patients, elevated total VZV‐binding antibodies exhibit altered specificity: they competitively bind viral glycoproteins (e.g., gH/gL), preferentially targeting non‐neutralizing epitopes rather than critical neutralizing domains. The epitope drift functionally impairs antibody‐mediated viral entry blockade, explaining the age‐associated diminishment of vaccine protection [473]. Furthermore, senescent T cells (PD‐1^+^ CD4^+^ T cells) secrete excess TNF‐α, triggering pathological calcium influx in dorsal root ganglion neurons that drives postherpetic neuralgia hyperalgesia [474]. Collectively, the unique pathogen–immunosenescence interplay heightens latent pathogen reactivation risk (e.g., Mtb, VZV) and amplifies chronic sequelae burden.

### 5.6 Chronic Obstructive Pulmonary Disease

COPD is a chronic inflammatory pulmonary disease characterized by persistent airflow limitation and lung tissue destruction, representing the third leading global cause of death. Pathogenesis of COPD is influenced by genetic and environmental factors (e.g., smoking) and is further modulated by underlying pulmonary pathologies, such as chronic bronchitis and systemic inflammation. Immunosenescence is now recognized as a pivotal driver accelerating COPD progression and exacerbation. Senescent immune cells exhibit broad functional impairment, which disrupts pulmonary immune homeostasis and host defense, thereby markedly increasing susceptibility to COPD and elevating risk of disease progression to severe stages and mortality [475]. As exemplified in multiple models, senescent macrophages and neutrophils downregulate expression of phagocytosis‐related receptors (e.g., CD206 scavenger receptors in macrophages, FcγRs in neutrophils), coupled with impaired fusion between phagosome and lysosome, leading to significant reduced clearance efficiency for bacteria or apoptotic cells [476]. Similarly, senescent B cells display defective antibody production, ultimately attenuating IgG and IgA responses against respiratory pathogens like influenza viruses and Streptococcus pneumoniae [477]. In COPD patients, abnormal accumulation of senescent T‐cell subsets (e.g., CD8^+^ KLRG1^+^ TEMRA T cells, CD28^−^CD57⁺ T cells) not only dampens immune clearance of respiratory viral infections but also depletes regenerative capacity.

Despite comparable exposure to risk factors, only a fraction of individuals develops into COPD, indicating that interindividual variation in inflammatory tone and autoimmune components governs disease susceptibility. The pulmonary inflammatory microenvironment promotes destruction of pulmonary parenchyma (e.g., alveolar wall collapse, airway remodeling, fibrosis, or mucus hyperplasia) and frequent acute exacerbation, thereby contributing to treatment resistance in COPD patients [478]. Within the pulmonary parenchyma of most COPD patients, a bunch of activated immune cells such as B cells, Th cells, and CD8^+^ T cells exhibit substantial elevations, whereas the frequency of anti‐inflammatory Treg cell subsets perform pronounced reduction. Formation of the unique immune horizon are regulated by immunosenescence particularly by SASP [479]. For example, senescent macrophages sustain proinflammatory gene transcription (e.g., IL‐8, TNF‐α) via histone deacetylase (HDAC)‐mediated epigenetic mechanisms, releasing abundant cytokines (e.g., TNF‐α, CXCL8) into the pulmonary niche. Moreover, neutrophil extracellular traps and ROS derived from senescent neutrophils stimulate secretion of IL‐1β in AMs, degrade pulmonary surfactant to amplify lung injury in COPD patients. Concurrently, senescent T cells overproduce MMP, exacerbating airway inflammation and promoting remodeling and fibrotic deposition, thereby propelling COPD pathogenesis [480].

### 5.7 Type 2 Diabetes Mellitus

Type 2 diabetes mellitus (T2DM) represents progressive loss of pancreatic β‐cell functional heterogeneity and insulin secretory capacity, frequently coinciding with insulin resistance and metabolic syndrome, thereby elevating morbidity risks of multisystem complications, including chronic kidney disease, hepatic disorders, and CVDs. According to *International Diabetes Federation* data, the global diabetic population reached 537 million in 2023, with China accounting for 141 million cases, imposing substantial public health and socioeconomic burdens [481, 482]. During early T2DM pathogenesis, compromised glucoregulatory homeostasis precipitates systemic metabolic dysregulation and chronic low‐grade inflammation, directly driving multidimensional immunometabolic imbalance [483]. Clinical evidence reveals significant expansion of senescent T cell populations in T2DM patients, concomitant with upregulated expression of senescence biomarkers (e.g., p16^INK4A^, CD27) [484]. Elevated senescent T cell demonstrates strong positive correlations with hyperglycemia and glycemic indicators (e.g., hemoglobin A1c, HbA1c), serving as prognostic indicators for deteriorating glycemic control [75, 485].

Within the pervasive reprogramming setting of T2DM, CD8⁺ T cells and macrophages exhibit multifaceted metabolic remodeling and switched into proinflammatory phenotype, characterized by enhanced lipid storage, mitochondrial ROS overproduction, and attenuated fatty acid β‐oxidation [486]. As a response, T cells undergo lineage remodeling to deplete naïve T‐cell and expand terminally differentiated effector memory T cells, as well as reduce TCR diversity; accompanying Th17/T imbalance promotes proinflammatory polarization, collectively driving T2DM‐associated immune dysregulation. Hyperglycemia‐induced impairment of efferocytic capacity in senescent macrophages precipitates intraislet accumulation of cytotoxic proteins (e.g., islet amyloid polypeptide), directly accelerating β‐cell exhaustion [487]. When confront with T2DM‐induced lipotoxicity and glucotoxicity, senescent macrophages secrete proinflammatory cytokines (e.g., TNF‐α) with overproduction of reactive ROS and NO, instigating maladaptive damage/stress responses that heighten diabetes susceptibility in geriatric cohorts [488]. Diverse SASP factors (IL‐6, TNF‐α, IL‐1β, IFN‐γ) derived from senescent immune cells exacerbate systemic chronic inflammatory burden, while inducing β‐cell apoptosis and suppressing insulin secretion, collectively propelling T2DM progression [489, 490].

T2DM‐driven metabolic dysregulation and glucolipotoxicity‐mediated oxidative stress substantially elevate the risk of chronic hepatic fibrosis and cirrhosis [485]. In the visceral adipose tissue of T2DM patients, senescent CD4⁺ and CD8⁺ T cells secrete TNF‐α, which impairs insulin receptor activation and serine residue phosphorylation of insulin receptor substrate 1, thereby increasing insulin resistance and markedly exacerbating the risk of hepatic fibrosis [490]. TNF‐α‐mediated proinflammatory signaling further activates hepatic stellate cells and drives excessive collagen deposition [491]. Building upon these mechanistic advances, the clinical translation of immunosenescence‐targeted interventions in T2DM is undergoing advancing refinement. Metformin, a first‐line cornerstone therapeutic agent for T2DM, beyond its well‐established classic antihyperglycemic actions, has also been reported to reduce senescent T cell burden and suppress their secretion of proinflammatory cytokines, thereby ameliorating hepatic inflammation and insulin resistance [492]. Targeted elimination of senescent immune cells (e.g., dasatinib plus quercetin) or blockade of SASP signaling (e.g., baricitinib inhibiting IL‐6 signaling) have also demonstrated significant improvements in insulin sensitivity and HbA1c reduction in clinical trials [493].

## 6 Immunosenescence‐Targeted Therapy

Both pathogenic conditions and therapies, especially in the case of tumor, can directly or indirectly induce widespread immunosenescence [388]. Blunted immune system during senescence fails to recognize or eliminate those abnormal cells, contributing to treatment resistance and unfavorable outcome. Preventing or reversing immunosenescent events at the source is supposed to restore antitumor immunity as well as broader immunological homeostasis and improve current limited therapeutic responses. Given the profound heterogeneity of immunosenescence, current therapeutic strategies are aimed to design targeted pharmacological agents, which exploits immunosenescent markers or and selectively clear extracellular SASP components. Furthermore, modulators of senescence‐associated autophagic flux and inflammatory signaling pathways offer complementary approaches to reprogram senescent cells and reverse their pathogenic phenotypes. Advancements related these immunosenescence‐targeted methods are combed and summarized in Table 5, while the ongoing clinical trials listed in Table 6.

**TABLE 5:** Innovative immunosenescence‐targeted therapeutic strategies.

Therapeutic strategy	Pharmaceutical	Diseases	Target cell	Mechanism	Function	References
Senescent immune cell reversal	AZD0156	Melanoma	CD4^+^ and CD8^+^ T cell	Inhibiting ATM phosphorylation	Inhibiting tumor growth	[494]
	U0126	Melanoma	T cell	Disrupting MAPK pathway	Inhibiting T cell senescence; increasing antitumor ability of T cell	[129]
	SP600125	/	T cell	Inhibiting AMPK–TAB1–p38 pathway	Reversing proliferative defect of senescent T cell	[495]
	iBFAR2	Bladder cancer; breast cancer; melanoma	CD8^+^ T cell	Inhibiting BFAR–JAK2–STAT1 signaling pathway	Restoring antitumor activity of senescent CD8^+^ T cell; inhibiting tumors growth	[23]
	Doxycycline	Triple negative breast cancer	CD8^+^ T cell	Decreasing expression of CD28	Inhibiting tumor growth	[496]
	Metformin	/	CD8^+^ T cell	Increasing content of telomerase; inhibiting expression of DDR‐genes	Decreasing number of senescent CD8^+^ T cell; inhibiting IFN‐γ secretion	[497]
	HCW9218	Pancreatic adenocarcinoma; prostate cancer	NK cell	Activating JAK3–STAT5 pathway; competitive binding of TGF‐β1/2/3	Promoting NK cell proliferation; reconfiguring antitumor activity of NK cell	[498]
	PGPC; WTLs; LPS	Melanoma	DC cell	Correcting defective migration of senescent DC cell	Driving antitumor immunity	[499]
	Sirolimus	Systemic lupus erythematosus	CD4^+^ and CD8^+^ T cell	Inhibiting mTOR signaling pathway	Increasing CD4^+^CD25^+^FoxP3^+^ regulatory T cell and CD8^+^ memory T‐cell populations	[500]
	Cinnomer	Multiple sclerosis	Dendritic cell	Increasing expression of HLA‐DR and CD86	Improving patient's symptoms	[501]
Senescent cell clearance	HDACi	Prostate cancer	Senescent neutrophil	Promoting elimination of senescent immunosuppressive neutrophils	Increasing efficacy of prostate cancer therapy	[58]
	ABT‐199	Pancreatic adenocarcinoma	Senescent CAF	Increasing proportion of activated CD8^+^ T cell	Decreasing tumor burden	[502]
	ARV‐825	Liver cancer	Senescent liver cancer cell	Recruiting BRD4 protein to CRBN; increasing degradation of BRD4	Promoting tumor cell apoptosis	[503]
	GSK3 inhibitor	Liver cancer	Senescent liver cancer cell	Inhibiting expression of PARP1 and AIF	Decreasing tumor burden	[504]
	ABT‐263	Systemic lupus erythematosus	Senescent CD4^+^ T cell; age‐related B cell	Inhibiting expression levels of BCL‐2 family	Decreasing autoantibody titers and kidney injury	[72]
	Dasatinib and quercetin	Alzheimer's disease	Senescent oligodendrocyte progenitor cell	Removing senescent cell from the plaque environment	Decreasing neuroinflammation; lessening Aβ load; ameliorating cognitive deficits	[282, 505]
	SSK1‐NP	Alzheimer's disease	Senescent N2a cell	Activating p38–p21 axis; inducing elimination senescent cell	Decreasing β‐amyloid protein accumulation	[506]
	Tie2–Cre system	Alzheimer's disease	Senescent endothelial cells and microglia	Decreasing tau phosphorylation	Improving spatial memory ability	[507]
	AP20187	Multiple sclerosis	Senescent microglia	Enhancing clearance of senescent cell; modulating inflammatory environment	Increasing formation of mature oligodendrocytes and completing myelination	[322]
	ABT‐737	Psoriasiform dermatitis	Senescent CD4^+^ T cell	Regulating TET2–Th17 cell pathway	Reducing severity of psoriatic lesions	[508]
	AP20187	Chronic ischemic renal disease	Senescent cell	Decreasing transformation of macrophages into myofibroblasts	Decreasing renal fibrosis	[509]
	EF24	Atherosclerosis	Senescent endothelial cell	Inhibiting Akt/mTOR/NF‐κB signaling pathway	Inhibiting atherosclerosis progression	[510]
Metabolic intervention	Mimic peptide	Colorectal cancer; melanoma	CD8^+^ T cell	Interrupting combination of accumulated lactate in TME and GLUT10	Promoting CD8^+^ T cell glucose utilization, proliferation, and antitumor functions	[511]
	Lithium carbonate	Colorectal cancer; melanoma	CD8^+^ T cell	Offloading lysosomal lactate to mitochondria by targeting on V‐ATP proton pump on lysosomal membranes	Promoting CD8^+^ T cell infiltration; inhibiting tumor growth	[512]
Senomorphic therapy	Apigenin	Breast cancer	CXCL10; IL‐6	Inhibiting NF‐κB activity	Inhibiting cancer cell proliferation	[513]
	Metformin	Head and neck squamous cell carcinoma	IL‐6	Inhibiting mTOR–STAT3–IL‐6 pathway	Inhibiting cancer stem cell populations	[514]
	HSP‐90 inhibitor	Malignant pleural mesothelioma	IL‐8	Inhibiting FAK–AKT signaling pathway	Inhibiting epithelial–mesenchymal transition	[515]
Simvastatin	Breast cancer	IL‐6	Inhibiting activation of Rac1 and Cdc42	Decreasing breast cancer cell proliferation and endocrine resistance	[516]
CCL11na	Multiple sclerosis	CCL11	Decreasing expression of myelin basic protein	Improving myelin regeneration	[322]
Immune function enhancement	B68	Colorectal cancer	Senescent colorectal cancer cell	Mediating ubiquitination‐mediated degradation of PD‐L1 by targeting CSN5	Promoting an antitumor immune response	[517]
	RPN1 siRNA	Melanoma	Senescent melanoma cell	Disrupting glycosylation and stabilization of PD‐L1	Restoring infiltration and killing ability of T cell	[518]

This table summarized current therapeutic strategies targeting immunosenescence, as well as listed the detailed methods, disease contexts, functional mechanisms, and effects.

*Abbreviations*: AIF, apoptosis‐inducing factor; AKT, protein kinase B; AMPK, adenosine monophosphate‐activated protein kinase; ATM, ataxia telangiectasia mutated; ATP, adenosine triphosphate; BRD4, bromodomain‐containing protein 4; CAF, cancer‐associated fibroblast; Cdc42, cell division cycle 42; CRBN, cereblon; CSN5, constitutive photomorphogenic 9 signalosome 5; CXCL10, C‐X‐C motif chemokine ligand 10; DC, dendritic cell; DDR, DNA damage response; FAK, focal adhesion kinase; GLUT10, glucose transporter 10; HDACi, histone deacetylase inhibitors; HSP‐90, heat shock proteins 90; ICB, immune checkpoint blockade; IFN‐γ, interferon‐γ; IL, interleukin; JAK2, Janus kinase 2; LPS, lipopolysaccharide; MAPK, mitogen‐activated protein kinase; mTOR, mammalian target of rapamycin; NF‐κB, nuclear factor kappa‐B; PARP1, poly (ADP‐ribose) polymerase 1; PD‐L1, programmed death ligand 1; PGPC, 1‐palmitoyl‐2‐glutaryl phosphatidylcholine; Rac1, Ras‐related C3 botulinum toxin substrate 1; SASP, senescence‐associated secretory phenotype; STAT, signal transducer and activator of transcription; TAB1, TGF‐β activated kinase 1/2‐binding protein 1; TGF‐β, transforming growth factor‐β; WTLs, whole‐tumor lysate.

**TABLE 6:** Clinical trials of immunosenescence‐targeted therapy.

Therapeutic strategy	Pharmaceutical	Stage	Diseases	Outcomes	NCT registration number
Reverse senescent immune cell	Doxycycline	Phase 3	Triple negative breast cancer	Inhibiting tumor growth	NCT02201381
	HCW9218	Phase 1/2 Phase 1 Phase 2	Advanced pancreatic carcinoma solid tumor ovarian cancer	Promoting NK cells proliferation; reconfiguring antitumor activity of NK cell	NCT05304936 NCT05322408 NCT05145569
	Sirolimus	Phase 1/2	Systemic lupus erythematosus	Increasing CD4^+^CD25^+^FoxP3^+^ regulatory T cell and CD8^+^ memory T‐cell populations	NCT00779194
	Glatiramer acetate	Phase 4	Multiple sclerosis	Improving patient's symptoms	NCT04928313
Senescent cell clearance	HDACi	Phase 2/3 Phase 1	Leukemia multiple myeloma	Increasing leukemia therapy efficacy increasing multiple myeloma therapy efficacy	NCT03564704 NCT01464112
	ABT‐199	Phase 1	Small lymphocytic lymphoma; chronic lymphocytic leukemia non‐Hodgkin's lymphoma	Decreasing tumor burden	NCT01682616 NCT01969695
	GSK3 inhibitor	Phase 1/2	Alzheimer´s disease	Decreasing neuroinflammation; lessening Aβ load; ameliorating cognitive deficits	NCT00948259
	ABT‐263	Phase 1	Lymphomas; leukemias solid tumors	Decreasing autoantibody titers and kidney injury	NCT00743028 NCT01009073
	Dasatinib and quercetin	Phase 1/2	Alzheimer's disease	Reducing neuroinflammation; lessening Aβ load; ameliorating cognitive deficits	NCT04785300 NCT04063124 NCT05422885 NCT04685590
Metabolic intervention	Lithium carbonate	Phase 1 Phase 2	Colorectal cancer familial adenomatous polyposis	Promoting CD8^+^ T cell infiltration; inhibiting tumor growth	NCT03153280 NCT05402891
Senomorphic therapy	Metformin	Early Phase 1 Early Phase 1 Phase 1 Phase 2	Head and neck squamous cell carcinoma psoriasis vulgaris non‐small cell lung cancer lymphoma	Inhibiting cancer stem cell populations inhibiting mTOR and RAS signaling pathways; promoting release of immune‐related factors by tumor cell and activate immune cell to exert antitumor immune effects blocking tumor growth by blocking a protein	NCT02083692 NCT02402348 NCT02325401 NCT01333852 NCT01031225 NCT01485536
	Simvastatin	Phase 2	Breast cancer	Decreasing breast cancer cell proliferation and endocrine resistance	NCT00334542 NCT00807950 NCT03324425
Immune function enhancement Immune escape suppression	B68	Phase 1 Phase 2	Relapsed/Refractory multiple myeloma Hodgkin's lymphoma; primary mediastinal large B‐cell lymphoma	Promoting an antitumor immune response	NCT05498545 NCT04875195

This table summarized clinical trials of immunosenescence‐targeted therapeutics, as well as listed detailed pharmaceutical agents, trial phases, disease indications, outcomes, and trial identifiers.

*Abbreviations*: DC, dendritic cell; HDACi, histone deacetylase inhibitors; HSP‐90, heat shock proteins 90; IL, interleukin; JAK, Janus kinase; mTOR, mammalian target of rapamycin; NF‐κB, nuclear factor kappa‐B; RAS, renin–angiotensin system; STAT, signal transducer and activator of transcription.

### 6.1 Targeted Blockade of Senescent Immune Cells or Senescence‐Related Signals

Among various senescent immune cell populations, senescent T cells demonstrate significant clinical relevance, especially strongly associated with poorer outcomes including tumor recurrence, disease progression, and increased mortality in cancer patients. Correspondingly, senescence of CD8^+^ T cells can also lead to excessive secretion of SASP and continuous development of chronic inflammation within TME, supporting tumor cell immune evasion and proliferation [519]. Consequently, therapeutic strategies aimed at reversing T cell senescence have emerged as a promising avenue to rejuvenate immune competence and restore effective immune responses across a broad spectrum of diseases (Table 5) [132, 145, 520].

Considering the leading role of abnormally activated ATM or MAPK signals in driving cycle arrest and senescence of CD4^+^ and CD8^+^ T cells, blocking ATM and MAPK signaling pathways is expected as viable option to reverse senescent T cells [129, 521]. Pharmacological inhibition of ATM phosphorylation in CD4^+^ and CD8^+^ T cells by specific inhibitors (e.g., KU55933) effectively reverse senescence of T cells [494]. Similarly, targeted disruption of MAPK pathway components—including phosphorylated p38, extracellular signal‐regulated kinases (ERK), and JNK—through specific inhibitors (LY2228820, U0126, SP600125) or genetic silencing techniques has shown comparable efficacy in rejuvenating senescent T cells and restoring their antitumor capacity [25, 129, 161, 495]. Moreover, the therapeutic benefits of these ATM and MAPK inhibitors extend beyond cellular rejuvenation. By alleviating the burden of immunosenescence, these compounds may potentiate the efficacy of conventional immunotherapies and chemoradiotherapy regimens [522, 523, 524]. In addition, other signaling pathways like mTOR, natriuretic peptide receptor 2 (NPR2)–cyclic guanosine monophosphate (cGMP)–protein kinase G (PKG) and p53 also play an active role in senescence of T cells. For example, using micelles for targeted delivery of rapamycin, mechanistic target of rapamycin complex 1 signaling pathway is inhibited, rejuvenating senescent T cells in tumor lymph nodes and enhancing efficacy of ICB [525]. NPR2–cGMP–PKG axis in CD8^+^ T cells is activated by inositol requiring enzyme 1 alpha (IRE1α) inhibitors, decreasing expression of aging biomarker KLRG1 and tumor burden [526]. During the aging process of T cells, p53 plays a positive role in cell cycle arrest. After knocking out p53 gene, the aging characteristics of CD8^+^ T cells was significantly reduced, such as the expression of SA‐β‐gal^+^. This enhances the ability of T cells to migrate to meningeal lymphatic vessels and also inhibits tumor growth in mice with breast cancer leptomeningeal metastasis [527].

In addition to targeting upstream modulatory signals, enhancing telomerase activity is supposed to mitigate T cells from cell cycle arrest and alleviate senescence [528]. Considering the pivotal role of CD28–IL‐2 signaling axis in regulating cell cycle of T cells, exogenously introducing CD28 gene augments telomerase activity and delays senescence of CD8^+^ T cells [529, 530, 531]. Treatment with metformin can upregulate content of telomerase in PBMCs isolated from different age populations by promoting production of IL‐2, inhibiting expression of DDR‐genes to restore antiaging ability [497]. Moreover, targeting ATM and MAPK pathways may reduce detrimental effects of senescent T cells on progression of autoimmune diseases (e.g., RA and giant cell arteritis) and CNS disorders (e.g., AD and PD), by preventing crucial inflammatory mediators neural involved in tissue damage or neurodegeneration [532, 533].

Rebalancing disordered metabolic functions of senescent T cells such as impaired glucose uptake, suppressed lysosomal and mitochondrial function offer another therapeutic avenue. Accumulated lactate in TME can bind to glucose transporter 10 (GLUT10) on CD8^+^ T cells to inhibit their glucose uptake, while interrupting the combination by mimic peptide effectively restores glucose uptake of CD8^+^ T cells, and prolongs survival of melanoma mice [511]. Intracellular lactate can also cause lysosomal acidification and damaged mitochondrial metabolism to promote senescence of CD8^+^ T cells. Through targeting on V‐ATP proton pump on lysosomal membranes, lithium carbonate offloads lysosomal lactate to mitochondria, thereby recovering those injured and senescent CD8^+^ T cells. In lung cancer or breast cancer mice models, lithium carbonate synergizes with PD‐1 antibody obviously prolongs survival time of tumor‐bearing mice, compared with PD‐1 antibody monotherapy [512]. Administration of lithium carbonate has also been observed to restore proliferative capacity and mitochondrial function of senescent T cells in RA, significantly alleviating inflammatory pathology, including reduced synovial inflammation, decreased levels of IL‐17 and TNF‐α, and attenuation of joint swelling and tissue damage [534, 535].

Besides with T cells, target elimination of other senescent immune cells (such as macrophages, DCs, and neutrophils) is also feasible to remit immunosuppressive microenvironment at different dimensions and improve comprehensive clinical benefits for patients [56]. For instance, selective depletion of senescent TAMs reduces Treg cells infiltration but expands intratumoral CD8^+^ T cells in NSCLC models, being conducive to inhibiting tumor growth and prolonging survival time of tumor‐bearing mice [57]. This therapeutic paradigm has been successfully replicated in other malignancies, including glioma and additional lung cancer models [56, 209]. Using senolytic drug fisetin to eliminate senescent macrophages can also improve function of muscle stem cells in Duchenne muscular dystrophy. Likewise, other senolytic agents such as ABT‐263 can significantly reduce number of senescent macrophages and microglia in experimental autoimmune encephalomyelitis (EAE), thereby alleviating severity of motor symptoms and promoting neuronal survival in EAE mice [88, 536]. Treatment with specific pharmacological inhibitors targeting senescent immune cells has also been evaluated. Cell superactivator toward DCs, 1‐palmitoyl‐2‐glutaryl phosphatidylcholine (PGPC) adjuvanted with whole‐tumor lysates and lipopolysaccharide, corrects defective migration of senescent DC cells, which also induces differentiation of CD4^+^ T cells into cytolytic Th1‐CD4^+^ T cells [499]. Compared with amplifying antitumor effect, senescent neutrophils clearance shows more obvious synergistic effect with other antineoplastic drugs. For example, clearing senescent neutrophils with HDAC inhibitors improves response to chemotherapy and ENZA (a standard prostate cancer treatment) [58]. Similarly, the cardiac glycoside ouabain exhibits synergistic antitumor effects when combined with chemotherapeutic agents in lung cancer models [537].

### 6.2 Inhibition of SASP‐Mediated Immunosuppression

The pivotal role of SASP underscores the promising value in clinical diagnostics and therapeutic interventions. However, source and compositional heterogeneity of SASP factors set the first obstacle in identifying disease‐specific biomarkers, while their spatial distribution heterogeneity makes it more embarrassed.

As previously described, SASP exhibits context‐dependent duality in tumor modulation, with effects largely determined by its cellular origin and molecular composition. Certain SASP components, including TNF‐α and ICAM‐1, positively induce and trigger immune responses including NK cell activation [343, 538]. Therefore, augmenting these antitumor SASP components may potentiate antitumor responses. Paradoxically, the majority of SASP mediators exhibit immune suppressive properties. Inhibiting or blocking these potentially protumorigenic SASP components is considered as a feasible strategy to suppress tumors or improve efficacy of existing therapies. Based on this, the concept of senomorphic therapy, aiming at converting immunosenescence by specifically blocking secretion of proinflammatory or protumorigenic SASP factors, has been proposed and tested in preclinical trials [539]. A subset of currently available drugs such as NF‐κB pathway inhibitors are adopted to disrupt regulatory elements to interfere synthesis of these SASP components and their biological effects in tumor proliferation [540]. For instance, in breast cancer, flavonoid drugs like apigenin have been confirmed to inhibit NF‐κB‐dependent production of CXCL10 in tumor cells, thereby reducing proliferation of precancerous epithelial cells and invasiveness of human breast cancer cells [513]. In clinical cohorts of HNSCC patients, metformin‐mediated inhibition of mTOR–STAT3 pathways reduces IL‐6 production and cancer stem cell populations, correlating with improved clinical outcomes [514]. Similarly, production of proinflammatory SASPs such as IL‐6 and IL‐8 is effectively curtailed by knocking out upstream regulatory molecules in HCC mouse models, which markedly attenuates tumor growth rates and progression compared with control groups [541].

In addition to their direct effects on tumor proliferation and malignant progression, inhibiting or eliminating protumorigenic SASPs is also reported to augment efficacy of current therapy. For example, heat shock proteins 90 inhibitors reduce circulating SASPs (particularly IL‐8) levels, thereby increasing chemosensitivity in malignant pleural mesothelioma [515]. In pancreatic cancer models, compared with ICB monotherapy, combination with Bcl‐2 inhibitors and ICB reduces secretion of SASPs by depleting senescent CAFs, significantly decreasing tumor burden in murine models [502]. A parallel therapeutic benefit has been observed in breast cancer, where simvastatin‐mediated suppression of senescent cell‐derived IL‐6 secretion mitigates chemoradiotherapy‐induced endocrine resistance, ultimately improving treatment outcomes [516].

Targeting SASP also demonstrates emerging therapeutic efficacy across various nononcological pathologies. Selectively blocking core signaling pathways (e.g., NF‐κB, p38/MAPK, mTOR) is supposed to suppress synthesis and secretion of proinflammatory SASP from the source, thereby mitigating pathological inflammation in diverse diseases. For example, rapamycin, a canonical inhibitor, selectively inhibits NLRP3 signals to reverse aberrant SASP production in different preclinical models, and ameliorates diabetes‐associated immunosenescence and vascular aging [542]. Novel small‐molecule compounds (e.g., SR12343, PX‐478, C25‐140) further attenuate SASP release while reducing senescence biomarkers in multiple tissues (liver, muscle, blood) [543]. Neutralizing antibodies against distinct SASP components such as IL‐6 have significantly improved clinical outcomes in age‐related disorders including RA.

### 6.3 Interference of Senescent Tumor Cells

Extensive preclinical evidence has demonstrated that there is a unique population of tumor cells that enter into senescent status during conventional anticancer therapies (e.g., radiotherapy and chemotherapy), which is defined as senescent tumor cells (STCs). The quiescent nature and acquired stemness of STCs endow them with diminished sensitivity to therapeutic stimuli, ultimately enhancing tumor aggressiveness and resistance. Principally, immune checkpoint molecules (e.g., PD‐L1/PD‐L2) are markedly stimulated and stabilized in STCs, participating in induction of cell‐intrinsic senescence when interacting with immune cells as well as systemic immunosenescence [518, 544, 545]. Blocking these immunosuppressive signals by ICBs effectively terminates senescent states of T cells and NK cells, reinstating their cytotoxic capacity to counteract immunosenescence. As initially reported in 2017, after anti‐PD‐1 therapy, 14 lung cancer patients exhibited varying degrees of “rejuvenation” phenomena including hair repigmentation [546]. Subsequent investigations validated that PD‐1 blockade facilitates immune‐mediated clearance of senescent cells, mitigating SASP‐associated diverse tissue inflammation [249]. In breast cancer models, PD‐L2 antibody significantly reduces STCs burden and reverses senescent phenotypes in T cells [547]. Targeted inhibition of PD‐1/PD‐L1 and other family members is emergingly regarded as a novel STCs‐eliminating strategy, with clinical value identified across multiple tumor types including melanoma, NSCLC, renal cell carcinoma, and bladder cancer [548]. Beyond inducing senescence in multiple tumor‐infiltrating immune cells (e.g., T cells, NK cells, macrophages), STCs also interact with cancer associated cells like fibroblasts and endothelial cells to exacerbate their protumorigenic effects [371, 539]. Therefore, therapeutic interventions aimed at STCs elimination or disrupting the “STCs–immunosenescence–tumor progression” vicious cycle represent a promising direction to reverse immunosuppressive microenvironment and reestablish antitumor immunity.

## 7 Prospects and Conclusions

Immunosenescence constitutes an inevitable, age‐associated deterioration of immune function, resulting from the cumulative impact of diverse environmental stressors. These extrinsic drivers dynamically trigger extensive immunoinflammatory and immunometabolic reprogramming, coupled with complex intracellular responses to perpetuate systemic aging. For instance, lifelong exposure to high antigenic loads (e.g., persistent viral infections or aberrant cells) exhausts antigen‐presenting capacity, while age‐related thymic involution constrains T‐cell output and repertoire diversity, thereby weakening immune surveillance against pathogens or malignant cells. However, disease‐specific stressor in dominating immunosenescence initiation remains not fully disclosed. As response to unrelenting stress, contributions of thymic degeneration and genomic instability in immunosenescence need to be delineated. Genetically heterogenous HSCs differentiation potential also serves as a deterministic factor in immune cell regeneration and immunosenescence onset.

Immunosenescence manifests as a multifactorial cascade wherein immune cell subtypes exhibit varying susceptibility and functional heterogeneity, becoming a major risk factor for diverse pathologies, including cardiovascular and neurodegenerative diseases. Notably, both neoplastic and non‐neoplastic conditions reciprocally accelerate immunosenescence through bidirectional pathological feedback. This bidirectional crosstalk is driven by the unique paracrine “contagion” effect of senescent immune cells, which reshape neighboring cells to amplify the immunosenescent signal via SASP‐mediated metabolic and secretory reprogramming. The dynamic and spatio‐temporal heterogenous nature of SASPs endow them with multifaced function, necessitating comprehensive and precisive mapping of cell‐type‐specific SASP profiles across diseases.

Distinct from isolated cellular senescence, development of immunosenescence is a protracted and highly dynamic process. Elucidating the multidimensional molecular signatures and spatiotemporal patterns of disease‐associated immunosenescence represents an urgent research priority. Recent advances in ML and AI now enable precise identification and assessment of cell‐type‐specific immunosenescent signatures across pathological contexts. By integrating multiomics data, spatial transcriptomics, and clinical parameters, multidimensional immunosenescence within germinal center, peripheral region, or pathologic niche are becoming distinct, facilitating predictions of therapeutic response and patient prognosis. However, significant phenotypic overlap exists between immunosenescence and other drivers of immune dysfunction such as T‐cell exhaustion necessitates expanded datasets to delineate its unique molecular architecture. Moreover, there remains significant gaps in revealing the core drivers and stage‐specific characteristics of immunosenescence in the complex temporal and spatial context. For instance, in the COVID‐19 infection models, monocytes exhibit accelerated but partially reversible aging phenotypes, highlighting context‐dependent dynamics of immune senescence.

Given the pivotal role of immunosenescence in the pathogenesis of numerous non‐neoplastic and neoplastic diseases, targeting immunosenescence is regarded as valuable therapeutic strategy. Senotherapies designed to eliminate senescent cells or block SASP signaling have emerged as promising interventions to ameliorate age‐related pathologies. Nevertheless, the precision and potential off‐target effects of specific approaches must be rigorously evaluated alongside their efficacy, to avoid possible biosafety issue. Immunosenescence‐targeted therapeutics are supposed to provide promising therapeutic targets and overcome resistance especially in the fields of degenerative disease and oncology, representing a frontier for next‐generation precisive treatment.

## Author Contributions

Ninghan Gong, Xiting Pan, and Yusi Deng conceived the structure of the manuscript, drafted the initial manuscript, and prepared the figures. Jiajia Che, Junhao Bao, and Mengqi Wang prepared the tables. Ying Shi, Xiaowei Liu, and Chuan Xu revised the manuscript. All the authors read and approved the final manuscript.

## Funding

This work was supported by grants from the National Natural Science Foundation of China (No. 82203539 to Ying Shi, No. 82473064, No. 22105137 to Xiaowei Liu), Natural Science Foundation of Sichuan Province (No. 2024NSFSC1919 to Xiaowei Liu), and National Key Research and Development Program of China (2022YFC2504700 [2022YFC2504703] to Xiaowei Liu).

## Ethics Statement

The authors have nothing to report.

## Conflicts of Interest

The authors declare no conflicts of interest.

## Data Availability

The authors have nothing to report.
